# Effect of ’Kuat’ a theory- and web-based health education intervention on mental health literacy among university students: A study protocol

**DOI:** 10.1371/journal.pone.0283747

**Published:** 2023-03-31

**Authors:** Siti Hafizah Zulkiply, Rosliza Abdul Manaf, Rahima Dahlan, Rahmat Dapari

**Affiliations:** Faculty of Medicine and Health Sciences, Universiti Putra Malaysia, Serdang, Selangor, Malaysia; Yokohama City University, JAPAN

## Abstract

**Background:**

Mental health problems, particularly depression and anxiety disorders are the leading causes of disease burden. Despite the effectiveness of mental healthcare services and the impairing effects of untreated mental health problems, the rate of help-seeking is low among young people. In addition, the mental health burden gap is high in low- and middle-income countries. Good mental health literacy has been associated with better help-seeking.

**Aims:**

This study aims to evaluate the effect of theory- and web-based health education intervention on mental health literacy among foundation students at a public university in Malaysia.

**Methods:**

A randomised controlled trial study will be conducted among foundation students. Participants will be recruited and randomly assigned to either the intervention or control group. The intervention will be conducted for two weeks with a one-month follow-up. The health education intervention will be developed according to the Information, Motivation, and Behavioural Skill Theory, and will be delivered via a website. The outcome will be measured using validated, self-administered questionnaires. at baseline, post-intervention, and one-month follow up. The data will be analysed using Generalised Estimating Equation (GEE). This study is registered to the Thai Clinical Trial Registry (TCTR) (reference number: TCTR20210705006), dated 4^th^ July 2021.

**Conclusions:**

**T**he results from this study will be useful for relevant authorities to take further efforts in mental health promotion among young people.

## Introduction

Mental disorders represent 60–70% of disability-adjusted life years (DALYs) among young people. Depression has been identified as a rapidly growing epidemic disease that is forecasted to be the leading cause of the global burden of disease in 2030 [1]. The highest incidence rate of depression is found during adolescence. The global prevalence of depression and anxiety among adolescents is reported to be 25.0% and 31.0%, respectively [2]. Globally, depressive disorders are ranked the single largest contributor to non-fatal health loss, while anxiety disorders are ranked as the sixth [3]. In addition, both depression and anxiety are frequently associated to be leading to suicidal ideation [4, 5].

Mental disorders among young people are often unrecognised and untreated. Once identified, only 1 in 5 children and adolescents with mental health problems receives a mental health treatment. Untreated mental illness and delayed help-seeking have many negative consequences. Adolescents with no intention to seek help were found to have significantly higher odds of having depression than those who seek help [6]. The remission rate of depression was significantly decreased with six months or more of untreated depression [7]. In addition, longer untreated depression was significantly associated with greater severity of depression [8]. In another study, untreated panic disorder patients for longer than 1 year were found to have a high frequency of comorbid major depressive disorder [9].

Delayed help-seeking and untreated mental illness among young people are caused by factors such as limited resources, individual barriers of social stigma, and poor health literacy [10]. However, mental health literacy has been proven as a key facilitator of formal help-seeking. Those who recognised mental health disorders were found to be three to four times more likely to take some actions [11]. People with positive mental health literacy were also characterised as being more likely to give proper advice to psychologically distressed friends, to have less stigma towards psychiatric illnesses, and to have positive views about mental health services [12]. In addition, positive mental health literacy was also associated with good mental health status [13, 14].

Considering the long-term impacts mental illness could have, it is beneficial for young people to have good mental health literacy. However, studies showed that young people have only a moderate level of mental health literacy [15, 16]. In a study among 1707 of 12 to 14 years old students, only 3.0% of them were reported to have adequate mental health literacy [6]. Similarly, the level of recognition of mental disorders in 325 students was found to be low, with 27.5% of them recognised anxiety and only 42.4% of them identified depression [11]. Although it was reported that students had better recognition of depression than recognition of anxiety disorder, the rate of recognising depression was still low [15, 17–19].

In addition, interventions to increase mental health literacy among young people are limited, as most of the previous studies were conducted among adults [20–23]. In view of that it is critical that the issue be tackled during this period (youth). Ideally, young people should be able to recognise mental health problems and seek help when needed. To address this issue, intervention studies that can cater to this specific population should be developed [24].

The mental health gap among young people in low- and middle- income countries (LMICs) is critical [25], with more than 80% of depressive disorders are reported in the countries [3]. Furthermore, it was reported that 73–93% of people with depression and 85–95% of people with anxiety were not covered by treatment in LMICs [26]. Intervention studies of mental health are also very limited in LMICs, as most of the studies were conducted in high-income countries [27]. Malaysia, which is one of Asian countries, is also a middle-income country. The stigma of mental health among Malaysians is high, thus leading to poor help-seeking.

However, despite the importance of promoting good mental health among young people, little empirical research attention is given and interventions for improving mental health among young people are not well established [28]. Also, it is important for the mental health education as a means of intervention to be developed and its effectiveness to be evaluated. Furthermore, most of the digital mental health interventions were developed in the Western countries, and the trials for these programmes were also conducted in the Western countries. On the other hand, such intervention and trials in other countries are lacking.

One of the recommended strategies for an effective health education intervention is by using a theory-based intervention [29–31]. Psycho-behavioural theory-based health education intervention allows an understanding of human behaviour. This study will develop a health education intervention based on the information, motivation, and behavioural (IMB) Theory. In addition, it was suggested that promotional healthy behaviours using psychoeducation and effective theory-based psychological interventions should be increased by leveraging technologies [32]. These technologies have become a part of everyday lives, and youths of 15–24 years old are identified as the most connected age group with the technologies. It was reported that 80% of youth possessed mobile phones, and one in three was Internet users [33]. Digital health interventions therefore appear to be a promising strategy for health education among youth. Therefore, in view of the ubiquitous technologies in young people with mental health issues, they seem to be beneficial to be incorporated in the interventions [34].

The objective of this study is to develop, implement and evaluate the effect of theory- and web-based health education intervention on mental health literacy among a group of youths who are the foundation students in a public university in Malaysia. A randomised controlled trial will be conducted to evaluate the effect on mental health literacy. By increasing mental health literacy, the healthcare facilities will be able to reduce mental health problems through early recognition and intervention. The findings in this study can bring great benefit to the mental health promotion programmes in Malaysia, especially among the youth.

The specific objectives of this study are to develop a theory- and web- based health education intervention on mental health literacy among foundation students, to determine and compare the baseline of sociodemographic characteristics and mental health literacy among foundation students between intervention and control groups, and to evaluate within and between group effects of a theory- and web-based health education intervention on mental health literacy at 2 weeks post-intervention and one month follow-up.

## Methods

### Clinical trial registry

This study is registered to the Thai Clinical Trials Registry (TCTR) (reference number: TCTR20210705006) dated 4^th^ July 2021.

### Study duration

The study will be conducted over a period of 24 months, from September 2021 until August 2023, with data collection commenced in September 2022. The duration of the study is comprised of planning of the research, development of the health education intervention programme, implementation of the programme, and evaluation of the programme.

### Study design

**Fig 1:**
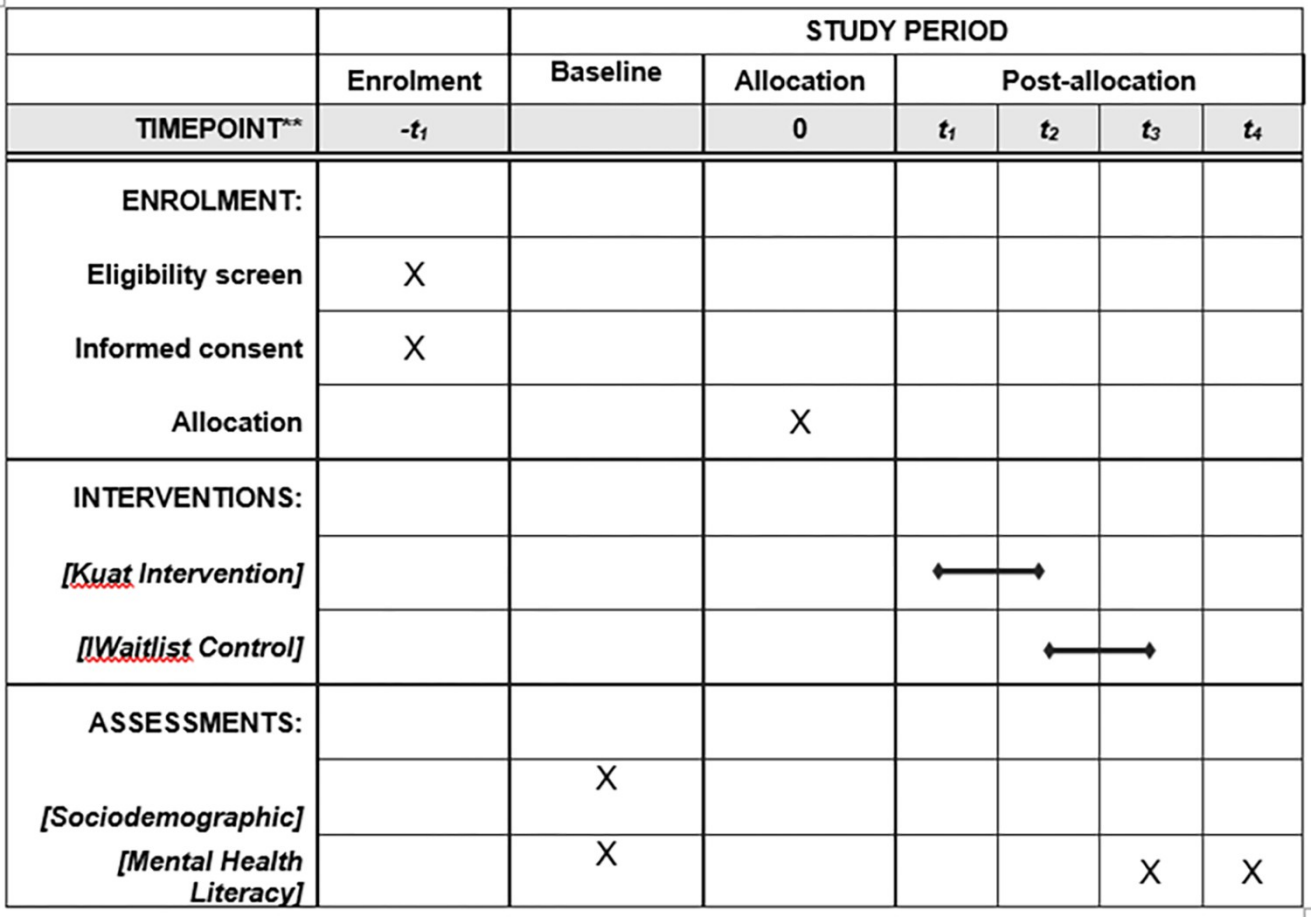
Schedule of enrolment, interventions, measurement, and assessment.

This study is a randomised controlled trial study with two arms involving an intervention and a control group. The participants who have consented to join the study will then be randomised to either the intervention group or the control group. The intervention group will be given a theory-based health education intervention via a newly developed website. Currently, there is no mental health literacy programme among university students in Malaysia. Thus, the control group will be waitlisted. The intervention will be conducted for two weeks. Baseline measurements including sociodemographic, anxiety and depression literacy, stigma, and attitude towards seeking professional help questionnaire will be taken prior to the intervention. The anxiety and depression literacy, stigma, and attitude towards seeking professional help questionnaire will be repeated after the intervention (2 weeks) and at follow-up (1 month). Fig 1 displays the schedule of enrolment, intervention, and measurement that will be used as well as the corresponding time of assessment.

### Study population

The study population will be foundation students in Universiti Putra Malaysia, a public university in Malaysia. The foundation program is a pre-university programme attended by school leavers in Malaysia, typically are young adults aged 18 to 19 years as a requirement for them to enter the bachelor’s degree programme. The students must meet all inclusion and exclusion criteria to be recruited in the study.

### Inclusion criteria

The inclusion criteria in this study are as follows:

- Malaysian citizen.
- Literate in Malay or English language.

### Exclusion criteria

There are no exclusion criteria for this study.

### Intervention

The health education intervention called, ‘KUAT’ had been developed based on a systematic review and guided by the IMB Theory. The content of the interventions comprise of symptoms of depression and anxiety, risk factors of mental disorders, available mental health services, pharmacological and non-pharmacological treatment for anxiety and depression, and self-help strategies. The structure of the health education intervention is described in Table 1.

**Table 1:** Structure of the health education intervention.

Component and Content of Health Education Material	Intervention Group	Control Group (waitlist)	Strategy Delivery	Method of Delivery
**Knowledge**	√	√	Website	Video
• **Symptoms of anxiety and depression**				Information
• **Risk factors of mental disorder**				
• **Professional help available**				
**Motivation**	√	√	Website	Video
• **Ability to recognise specific disorder**				Information
Behavioural Skills	√	√	Website	Video
• **Attitude towards mental disorder**				Information
• **Attitude towards seeking help**				
• **Self-help intervention**				

Based on the content, a total of five modules have been developed. Module 1 focuses on mental disorders among young people. Module 2 focuses on epidemiology and risk factors of mental disorders. Module 3 focuses on signs and symptoms of anxiety and depression, as well as screening tools for anxiety and depression. Module 4 focuses on available mental healthcare services in Malaysia, as well as treatments for anxiety and depression. Finally, Module 5 focuses on self-help strategies especially in managing stress.

Multi-professional experts including psychiatrists specialised in adolescents and public health physicians experienced in health education intervention of mental health literacy among young people have reviewed the modules for content and face validity. The modules have also been reviewed by university students. In each module, there will be a video, and information pertaining to the content and will be delivered via a website. Each video ranges from 3 to 5 minutes. The intervention will be given in two sessions over two weeks.

### Data collection

**Fig 2:**
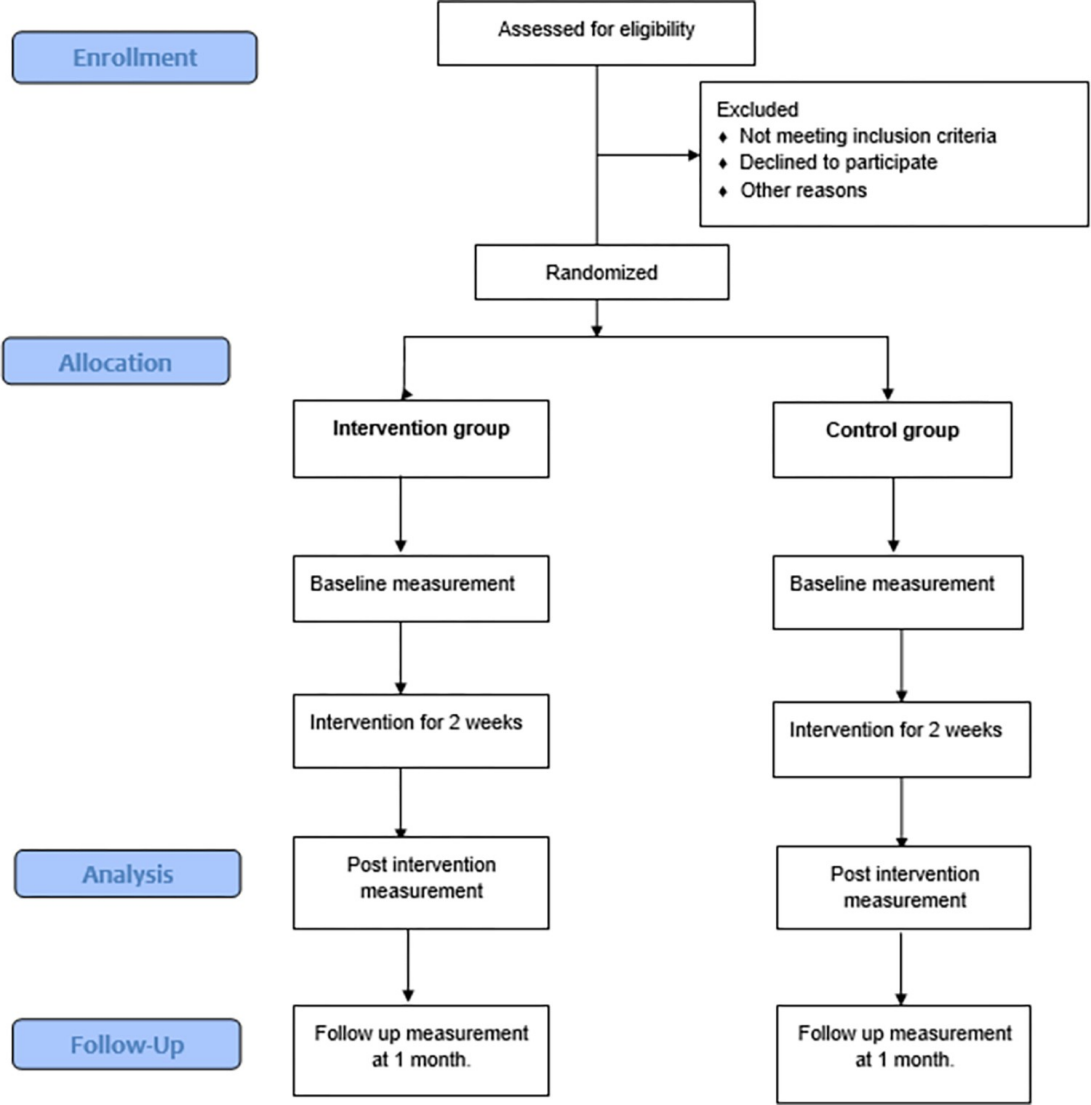
Flow diagram of study conduct based on CONSORT statement.

Data will be collected using pre-tested questionnaires. Evaluations will be made at three-time points: first at the baseline after consent is taken from the participants, second in the following two weeks after the intervention and finally at one-month follow-up. The target audience will be invited to join a WhatsApp group. Through the group, information regarding the study’s objectives and activities will be informed. Subsequently, a link to a consent form will be given. Those who have consented will be contacted, baseline measurement will be taken. After that, those in the intervention groups will be given the link to the intervention website with a respective encrypted username. Those who have not viewed and completed the module will be reminded. Finally, participants will be given the same set of questionnaire at 2-weeks post- intervention and at 1-month follow-up. The flow diagram of the study conduct based on the CONSORT 2010 statement [35] is shown in the Fig 2.

### Outcome measurement

The primary outcome for this study is mental health literacy. Guided by the IMB Theory, a set of questionnaires will be employed. Knowledge changes refer to changes in total knowledge score as measured in the questionnaire, which assesses the respondents’ level of knowledge on symptoms and risk factors of anxiety and depression and professional help available. To measure the knowledge and motivation, depression literacy (D-Lit) [36, 37] and anxiety literacy (A-Lit) [36] are adapted. The internal consistency of D-Lit and A-Lit is reported to be α = 0.70 and α = 0.76, respectively. The internal consistency of D-Lit among adolescents in Malaysia is reported to be α = 0.831 [38]. Both depression literacy and anxiety literacy consist of 22 items, respectively. The scale is measured by ‘true’, ‘false’, or ‘do not know’. One point is given for each correct response, and higher literacy is indicated by higher scores.

Behavioural skills score changes refer to self-efficacy components in the questionnaire whereby the score calculated is based on the ten statements. The attributes are openness to seeking treatment for emotional problems and values and need in seeking treatment. It will be measured by adapting to the Attitudes Towards Seeking Professional Psychological Help Scale-Short Form (ATSPPH-SF) [39] and Reported and Intended Behaviour Scale (RIBS). The internal consistency of ATSPPH-SF is reported to be α = 0.77. The factor analysis of ASPPH-SF reveals three dimensions namely “openness to seeking professional help”, “value in seeking professional help”, and “preference to cope on one’s own” [40]. The measure consists of 10 items and is rated using ‘agree’, ‘strongly agree’, ‘disagree’ or ‘strongly disagree.

Data obtained from a group of 30 respondents who fit in the inclusion and exclusion criteria will be used to determine the reliability of the questionnaire. The respondents were selected using convenience sampling. The questionnaires were distributed using Google Form and distributed through social media. The data collected were analysed to check for internal consistency of the questionnaires. Cronbach’s alpha coefficient values was used to determine the reliability of the questionnaire. The Cronbach’s alpha is 0.723, which indicates an acceptable value of reliability.

### Sample size

The sample size (n1) for this study was calculated by using the two population mean formula for hypothesis testing [41]. The sample size calculated was based on a study of effectiveness of a website intervention study on depression literacy [42]. The study had an effect of 0.54. To detect this effect with 80% power at the 0.05 significance level (one-tailed), the total sample size needed in each group (n_1_) in this study is 41. After considering a 50% loss to follow-up, a final sample size of 122 (61 respondents per arm) have been determined to be sufficient for this study.

### Randomisation

Participants who have given consent will be given a unique identification (ID) number. They will then randomly be allocated to the intervention or control group using their ID number. A randomised block design with a block size of four will be used, with the allocation of participants to the group established before the study is commenced [43]. The randomisation will be conducted by a person who is not a part of the research team, to ensure allocation concealment.

### Blinding

Single blinding will be applied involving the participants. The respondents will not be aware of the intervention allocation, but the researcher and those involved in providing the intervention module will be aware of the allocation. Blinding will be ensured by using an encrypted username for the participants. The participants will also be reminded not to share the content of the intervention.

### Data analysis

Data will be analysed using the IBM Corp. Released 2017. IBM SPSS Statistics for Windows, Version 25.0. Armonk, NY: IBM Corp. The data will be analysed according to the intention-to-treat analysis. Descriptive statistics will be used to describe the baseline characteristics of respondents. Mean and standard deviation will be used for continuous data, the median and interquartile range for non-normally distributed continuous data, and frequency and percentage for categorical data. The normality of the data will be checked using a histogram, Kolmogorov-Smirnov, and Shapiro-Wilk normality test.

The outcome variable will be compared at the baseline and post-test. For within-group difference, if data are assumed to be normally distributed data, parametric tests such as paired t-test will be utilised. But, if data are assumed to be not-normally distributed, non-parametric tests such Wilcoxon signed ranks test will be utilised. For between groups differences, if the data are assumed to be normally distributed, parametric tests such as independent t-test will be utilised. But if data are assumed to be not-normally distributed, non-parametric tests such as the Mann-Whitney U test will be utilised. Chi-square test and Fisher exact will be used to test the association between two categorical variables.

Multivariable analysis will be done using generalised estimating equations (GEE) to determine the effect of the intervention on the primary and secondary outcomes after adjusting for the covariates, including gender, status of family income, personal and family history of mental disorders, and history of usage in mental healthcare services. The effectiveness of the intervention will be based on the trial group and timepoint interaction result. The effect of the intervention on changes in outcome measures will be determined after the intervention period.

### Data management

Online questionnaires will be used for pseudonymised data collection. Study data and personal information will be stored in the using Microsoft excel. These data will then be transferred to the records and documentation system under the Universiti Putra Malaysia’s system. The data will then be destroyed five years later. Further to this, any study participant who have requested to know the study findings will be informed via an email correspondence.

### Study ethics

Ethical clearance has been obtained from the Ethics Committee for Human Research of the Faculty of Medicine and Health Sciences, Universiti Putra Malaysia (JKEUPM 2021–275). Informed consent will be obtained from the respondents prior to data collection digitally. Those who are interested after being briefed regarding study’s objectives and activities, will be given a link to study’ consent form. They will be required to click on the ‘agree’ button on the consent form link. There is no obvious risk by participating in this study. All participants will also receive information on help-seeking information, if deemed needed. Participants will be informed that they are allowed to withdraw at any time during the study period.

## Discussion

The aim for this study is to evaluate the effect of theory- and web-based health education intervention on mental health literacy among foundation students at a public university in Malaysia. Reviews have reported that mental health literacy interventions have been shown to increase literacy, particularly on anxiety [22, 44]. In consequence, improvement in mental health literacy will lead to an increase in help-seeking behaviour. Good mental health literacy has also been associated with good mental health status [13, 14]. Therefore, promotional interventions should include those that support children and young people to develop skills to maintain their mental health, and those that target pre-clinical risk factors, or distress response to early signs of distress.

In 2018, there were about a 5.5 million adolescents in Malaysia [45]. Youth is a crucial developmental period. First, during this period, brain development and maturation occur through dynamic and highly complex neural remodelling, involving changes in structures and connectivity. Neurodevelopmental changes that occur make it a period of both vulnerability and opportunity for mental health promotion. Social and developmental turmoil occurs during this period as youths are trying to negotiate several challenges, including transition into multiple social roles from the limited and dependent roles of childhood and simultaneous formation of distinct identities. Second, the period of youth is the starting point of overall mental health issues, as 50% of the onset of mental illness occurs at 14 years old and 75% occurs before 24 years old. Finally, mental health problems during youth have many consequences in later life. Young people are therefore, thought to be an important agent of change due to, the high prevalence of mental illness, the transitional phase to become an adult and the negative effects mental health problems could bring to their adult life.

The essential principles of improving solutions for mental health issues for young people need to be based on addressing barriers to accessing mental health services. This is in view of delays in treatment that will result in poorer outcomes [46]. About 25–50% of mental illnesses among adults may be prevented through early interventions during childhood and adolescence [47]. In addition, the economic benefit of early childhood interventions has been estimated on average to exceed their costs by a ratio of 1:6. The intervention from this study is therefore, thought to provide a valid and useful tool for mental health promotion.

The current pandemic of COVID-19 has precipitated an increase in depressive and anxiety symptoms among young people. This is either due to the pandemic itself or unprecedented interruption in their daily lives [48]. Due to the lockdown that has been taken as an action to control the pandemic, there is an even more pressing need for digital mental health intervention [49].

As the intervention from this study will be developed based on both literature review and theory, and as it will be delivered via website, the intervention would be beneficial for the target population. However, the mode of delivery (via website) would also cause some limitation to the study. It may possibly cause some loss to follow-up. These will be minimised with the collaboration with the lecturers and reminders to the students. Another limitation would be the study population, which is limited to one public university in Malaysia, which may affect the generalisation of the results.

Studies on mental health literacy intervention are limited in LMICs, including Malaysia. To the best of our knowledge, there is only one mental health literacy interventional study that has been conducted among adolescents in Malaysia [38]. Thus, the results from this study would be informative for further promotional activities among young people in Malaysia.

### Dissemination of results

Results from this study will be published in peer-reviewed international journals and presented at national and international conferences. The intervention website can be sustained and provide support for the national population if the intervention deemed efficacious.

## Supporting information

S1 File SPIRIT 2013 checklist: Recommended items to address in a clinical trial protocol and related documents. (DOC)

S2 File World Health Organization trial registration data set. (DOCX)

S3 File (PDF)
