# Supplementary material for: Effect of ’Kuat’ a theory- and web-based health education intervention on mental health literacy among university students: A study protocol
Source: PLoS One. 2023 Mar 31;18(3):e0283747. doi: 10.1371/journal.pone.0283747 (PMC10065237; doi:10.1371/journal.pone.0283747)
Supplement: S2 File — (DOCX) [file pone.0283747.s002.docx]

Table 1 World Health Organization Trial Registration Data Set

| Data category | Information |
| --- | --- |
| Primary Registry and Trial Identifying Number | Thai Clinical trial registry (TCTR20210705006). |
| Date of Registration in Primary Registry | 4^th^ July 2021 |
| Source(s) of Monetary or Material Support | N/A |
| Primary Sponsor | N/A |
| Contact for Public Queries | Dr Siti Hafizah Binti Zulkiply, hafizahzulkiply.hz@gmail.com |
| Contact for Scientific Queries | Dr Siti Hafizah Binti Zulkiply, hafizahzulkiply.hz@gmail.com |
| Public Title | Effect of a Theory-Based and Web-Based Health Education Intervention on Mental Health Literacy Among Foundation Students at UPM |
| Scientific Title | Effect of a Theory-Based and Web-Based Health Education Intervention on Mental Health Literacy Among Foundation Students at UPM |
| Countries of Recruitment | Malaysia |
| Health Condition(s) or Problem(s) Studied | Mental Health Problem |
| Intervention(s) | Theory based and web-based intervention on Mental Health Literacy |
| Key Inclusion and Exclusion Criteria | The inclusion criteria in this study are:  i. Malaysian citizen.  ii. Basic skills using digital or computer and internet literate.  iii. Have good command and understanding of the Malay and English language.  The exclusion criteria in this study are:  i. Currently receiving professional assistance for psychiatry diagnosis.  ii. Current severe self-harm or suicidal ideation. |
| Study Type | Randomized controlled trial |
| Date of First Enrollment | 1^st^ of October 2022 |
| Sample Size | 132 |
| Recruitment Status | Recruitment planned |
| Primary Outcome(s) | Mental Health Literacy |
| Key Secondary Outcomes | None |
| Ethics Review | Ethics Committee for Human Research of the Faculty of Medicine and Health Sciences, Universiti Putra Malaysia (JKEUPM 2021-275). |
