# Supplementary material for: Effect of ’Kuat’ a theory- and web-based health education intervention on mental health literacy among university students: A study protocol
Source: PLoS One. 2023 Mar 31;18(3):e0283747. doi: 10.1371/journal.pone.0283747 (PMC10065237; doi:10.1371/journal.pone.0283747)
Supplement: S3 File — (PDF) [file pone.0283747.s003.pdf]

EFFECT OF A THEORY-BASED AND WEB-BASED HEALTH EDUCATION INTERVENTION ON MENTAL HEALTH LITERACY AMONG FOUNDATION STUDENTS IN UPM

By

SITI HAFIZAH BINTI ZULKIPLY

FACULTY OF MEDICINE AND HEALTH SCIENCES

UNIVERSITI PUTRA MALAYSIA

2021

Approval Sheet

This thesis was submitted to the Faculty of Medicine and Health Sciences and has been accepted as fulfilment of the requirement for the Doctorate of Public Health. The members of Supervisory Committee were as follows:

Prof Madya Dr Rosliza Abdul Manaf

Department of Community Health

Faculty of Medicine and Health Sciences

University Putra Malaysia

Dr Rahima Binti Dahlan@ Mohd Shafie

Psychiatry Department

Faculty of Medicine & Health Sciences

Universiti Putra Malaysia

Dr Rahmat Dapari

Department of Community Health

Faculty of Medicine and Health Sciences

University Putra Malaysia

Declaration by Members of Supervisory Committee

This is to confirm that:

- The research conducted and the writing of this thesis was under our supervision;
- Supervision responsibilities as states in the Universiti Putra Malaysia (Graduate Studies) Rules 2003 (Revision 2012-2013) are adhered to.

Signature: ________________________

Name of Chairman of Supervisory Committee: Prof Madya Dr Rosliza Abdul Manaf

Signature: ________________________

Name of Member of Supervisory Committee: Dr Rahima Binti Dahlan@ Mohd Shafie

Signature: ________________________

Name of Member of Supervisory Committee: Dr Rahmat Dapari

TABLE OF CONTENTS

			Page
**CHAPTER**			
**1**	**INTRODUCTION**		
	1.1	Background of the Study	1
	1.2	Problem Statement	4
	1.3	Significance of Study	8
	1.4	Research Questions	12
	1.5	Research Objective	12
		1.5.1 General Objective	12
		1.5.2 Specific Objective	12
	1.6	Research Hypotheses	13
**2**	**LITERATURE REVIEW**		
	2.1	Burden of Mental Disorder	14
	2.2	Common Mental Disorder	15
	2.3	Determinants of Mental Disorder	18
	2.4	Impact of Mental Disorder	19
		2.4.1 Adult and Social	20
		2.4.2 Physical Health	20
	2.5	Management of Mental Disorder	20
		2.5.1 Psychotherapy	21
		2.5.2 Pharmacological	22
		2.5.3 Physical Activity	22
	2.6	Mental Health Literacy	23
		2.6.1 Factor Associated with Mental Health Literacy	26
	2.7	Help-seeking	26
		2.7.1 Factors Associated with Help-seeking Behaviour	27
	2.8	Assessment of Mental Health Literacy	30
	2.9	Health Behavioral Theories	31
		2.9.1 Social Cognitive Theory	31
		2.9.2 Health Belief Model	32
		2.9.3 Theory of Planned Behavior	32
		2.9.4 Information Motivation Behavioral Theory	33
		2.9.5 Theoretical Framework	34
	2.10	Digital Intervention	35
	2.11	A review on Health Education Intervention to Increase Mental Health Literacy	36
		2.11.1 Search Strategy	37
		2.11.2 Search Results and Baseline Characteristics	37
		2.11.3 Intervention	38
		2.11.4 Outcome Measure and Outcome	49
		2.11.5 Summary of Review on Interventions	45

2.12 Conceptual Framework 48

3 MATERIALS AND METHODOLOGY

3.1 Study Location 50
3.2 Study Duration 50
3.3 Study Design 50
3.4 Sampling Method 52
3.4.1 Study Population 52
3.4.2 Sampling Population 53
3.4.3 Selection Criteria 52
3.4.4 Sampling Frame 52
3.4.5 Sampling Unit 52
3.4.6 Sampling Method 52
3.4.7 Randomization 53
3.4.8 Blinding 53
3.4.9 Sample Size 53
3.5 Study Variables 55
3.5.1 Dependent Variables 55
3.5.2 Independent Variable 55
3.5.3 Covariates 55
3.6 Operational Definition 55
3.7 Study Instruments 56
3.7.1 Questionnaire 56
3.7.2 Health Education Intervention 56
3.8 Quality Control of Study Instruments 61
3.8.1 Questionnaire 61
3.8.2 Health Education Intervention 61
3.9 Data Collection 61
3.10 Data Analysis 63
3.11 Study Ethics 63
3.11.1 Ethical Approval 64
3.11.2 Consent 64
3.11.3 Data Storage 65
3.11.4 Publication 65
REFERENCES 66
APPENDICES 84

LIST OF TABLES

Table	Page
2.1 Diagnositic Criteria for Depressive Disorder	16
2.2 Diagnostic Criteria for Generalized Anxiety Disorder	17
2.3 Baseline Characteristics of Studies	40
2.4 Characteristics of Interventions	41
2.5 Outcome Measure and Outcome	44
3.1 Summary of Sample Size Calculation	54
3.2 Operational Definition	55
3.3 Structure of the Health Education Intervention Program	59
3.4 Summary of Statistical Tests	64

LIST OF FIGURES

Figure	Page
2.1 Mental Health Literacy Framework	25
2.2 Theoretical Framework	34
2.3 Conceptual Framework based on Information Motivation and Behavioral Theory	49
3.1 Flow Diagram of study Conduct based on CONSORT statement	51
3.2 Steps of the Development of Health Education Intervention Module	59

CHAPTER 1

INTRODUCTION

This chapter describes the background of mental health among young people. This chapter also outlines the problem statement, the significance of the study, the research questions, the research objectives, and finally the formulated hypotheses.

1.1 Background of the study

Youth (10-24 years old) is a time of substantial change both physiologically and psychologically (World Health Organization, 2021). Mental health problems usually emerged for the first-time during youth. Fifty percent of adult mental disorders have their onset before the age of 14 years, and 75% before the age of 24 years old (Kessler et al., 2007).

Mental health problems are most prevalent among adolescent, and it was estimated that 10-20% of adolescents experience mental health condition (World Health Organization, 2019a). Whereas, the 12 months prevalence of any mental disorder among people in their twenties was reported to be 19.8% among men, and 32.4% among women (Gustavson et al., 2018). Similarly, based on the National Health and Morbidity Survey (NHMS) 2015, the prevalence of mental illness among 5-15 years old in Malaysia was reported to be 12.1% (Institute for Public Health Malaysia, 2015). The same trend was reported in the NHMS 2019 where the prevalence of mental illness among 10-15 years old was 9.5% (Institute for Public Health Malaysia, 2019).

Both anxiety disorders and depression which were also referred to as a common mental disorders are the most prevalent psychological disorders in young people (Polanczyk et al., 2015). A meta-analysis reported a pooled anxiety prevalence of 19.1% in 19 studies, and a pooled depression prevalence of 14.3% in 36 studies (Barker et al., 2019). In Malaysia, one in 5 adolescents were reported to be depressed and 2 in 5 were reported to be anxious (Institute for Public Health, 2017).

According to the World Health Organization (WHO), mental health conditions in adolescent accounts for 16% of the global burden disease and injury (World Health Organization, 2019a). Mental health disorders are associated with disabilities as they can cause prolonged psychosocial, educational and occupational impairments (Esch et al., 2014; Kessler, 2012). The major impact of depression and anxiety is that they increases the risk of suicide or self-harm (Ahmad et al., 2014; Im et al., 2017). Suicide was reported to be the second leading cause of death among young people (Abbafati et al., 2020).

The trajectories of mental health illness can be altered through early recognition and intervention. Unfortunately, despite the detrimental and long-term effects of untreated mental health problems and the clear benefits of formal help, help-seeking among young people is at a troubling low rate. Besides, youths prefer to seek help from their families or friends rather than from professionals (Coles et al., 2016; Sarbhan Singh et al., 2019; Thai et al., 2020). Main barriers to help-seeking include poor mental health literacy and stigma. However, previous study reported only a moderate level of knowledge about depression among university students in Malaysia (Khan et al., 2010). Health education intervention would be a crucial step to improve mental health

literacy, stigma, and beliefs towards mental health services (Jung et al., 2017). Accordingly, health education intervention that aims to increase mental health literacy may improve help-seeking among the target group (Bohon et al., 2016; Jung et al., 2017).

The health education intervention for this study will be developed according to the information motivation behavioral (IMB) theory. The IMB theory constitutes that health-related information, motivation, and behavioral skills are fundamental determinants of health behavior. Mental health literacy is defined as the ability to recognize specific disorders, knowledge on risk factors, cause, self-treatment, and professional help available, and attitudes that promote recognition and appropriate help-seeking (A. F. Jorm, 2000). The IMB theory suits the components of mental health literacy framework. Therefore, adopting the IMB theory for health education intervention will foresee the increase in mental health literacy and ultimately, help-seeking behavior (Bohon et al., 2016).

Practically, the health education intervention for this study will be delivered by leveraging technology. Digital health interventions (DHIs) have frequently been highlighted as one way to respond to increasing levels of mental health problems in children and young people (Bergin et al., 2020). Focus group interviews among adolescents revealed that they were interested in receiving online health promotion for mental illnesses (Aschbrenner et al., 2019). Digital psychological intervention for mental health problems has also been recommended by experts from the United Nation (UN) to provide necessary mental support. Therefore, this study will be utilizing a digital platform as a medium for the health education intervention through a website.

Early recognition and appropriate help-seeking will only occur if young people know about early changes produced by mental health disorders, the best types of help available, and how to access this help (Kelly et al., 2007; Okello et al., 2014). Unfortunately, despite having substantial mental health gap evidenced by the high prevalence of suicidal attempt among young people in Malaysia (Malaysian Healthcare Performance Unit, 2017), the interventions to increase mental health literacy among young people in Malaysia is limited. Furthermore, Malaysian students were reported to have higher mental health problems, negative mental health attitudes, and lower self-compassion and resilience compared to UK students (Kotera et al., 2021). In view of, this research will develop a health education intervention adopting the IMB theory and will be delivered by leveraging technology, the findings from this study can be of great benefit for mental health promotion strategies among young people. This research project aims to evaluate the effectiveness of theory- and web-based health education intervention on mental health literacy among young people.

1.2 Problem Statement

Mental disorders, particularly depression, as a cause of disease burden is a widely quoted result of the Global Burden of Disease (GBD) study (Abbafati et al., 2020; S. L. James et al., 2018). Depression have been identified as a rapidly growing epidemic disease that forecasted to be the leading cause of the global burden of disease in 2030 (World Health Organization, 2004). It was estimated that major depressive disorders contributed to 50 million years lived with disability (YLD) (World Health Organisation, 2017).

Mental disorders particularly depression was the leading cause of disease burden in terms of YLD and the sixth leading cause of disability adjusted live years (DALY) among young people, posing a serious challenge to health systems (Abbafati et al., 2020). Anxiety disorders and depression are the most prevalent psychological disorders among young people (Polanczyk et al., 2015), with global prevalence of 25.0% and 31.0% respectively (Silva et al., 2020). Besides, there is a significant increase in 12 months prevalence of major depressive episode over the years from 2005 to 2014, among young people in the US aged 18 to 25 (Mojtabai et al., 2016).

Among the key risk factors contributing to suicidal behavior among youth were mental disorders (Bilsen, 2018). Suicidal behavior includes suicidal ideation, suicide attempts, and completed suicide. Both depression and anxiety were frequently associated leading to suicidal ideation (Ahmad et al., 2014; Im et al., 2017). Young people with a major depressive disorder are at seven times greater risk to have suicide. Suicide accounts for 9.1% of deaths and the third leading cause of mortality in 15-19 years old (Wasserman et al., 2005). Similarly, high suicidality among teenagers in Malaysia was reported. The NHMS 2017 reported a prevalence of suicidal attempts of 6.9% and 7.0% in girls and boys respectively (Institute for Public Health, 2017). In another study, the prevalence of suicidal ideation was 27.9% among 1769 secondary school students (Normala Ibrahim et al., 2017). Furthermore, the trend of suicide among young people is increasing.

Malaysia is one of Asia’s countries and is listed as a high middle-income country. The mental health gap among young people is intensified in low- and middle- income countries (LMICs) (Patton & Temmerman, 2016). More than 80% of depressive

disorder occurred in LMICs (World Health Organisation, 2017). Furthermore, it was reported that 73-93% of people with depression and 85-95% of people with anxiety are not covered by treatment in LMICs (Fu et al., 2020). Intervention studies of mental health are also very limited in LMICs, as most of the studies were conducted in high-income countries (Salam et al., 2016). In addition, stigma of mental illness is high in Malaysia, and an important predictor of help-seeking (Norhayati Ibrahim et al., 2019). This will then lead to the practice of seeking alternative care through spiritual practitioners, which is common among Malaysian (Raaj et al., 2021). Thus, it is crucial that awareness regarding mental illness in term of education is provided.

In addition, the current pandemic of COVID-19 has precipitated an increase in depressive and anxiety symptoms among young people. This is either due to the pandemic itself or unprecedented interruption in their daily lives (Nicole Racine, Jessica E. Cooke & Daphne J. Korczak, BraeAnne McArthura, 2020). Due to the lockdown that has been taken as an action to control the pandemic, there is an even more pressing need for digital mental health intervention (Shweta Singh et al., 2020).

Mental disorders among young people are often unrecognized and untreated. Once identified, only 1 in 5 children and adolescents with mental health problems received mental health treatment. Untreated mental illness or delayed help-seeking has many negative consequences. Adolescents with no intention to seek help were found to have significantly higher odds of having depression (Sarbhan Singh et al., 2020). The remission rate of depression was significantly decreased with six months or more of untreated depression (Bukh et al., 2013). In addition, longer untreated depression was significantly associated with greater severity of depression (Hung et al., 2017). In

another study, untreated panic disorder patients for longer than 1 year had a higher frequency of comorbid major depressive disorder (Altamura et al., 2005).

Delayed help-seeking or untreated mental illness among young people is owing to either limited resources, individual barriers of social stigma, or poor health literacy (Henderson et al., 2013). Mental health literacy has been proven as a key facilitator to formal help-seeking. Those who recognized mental health disorders were found to be three to four times more likely to take some action (Olsson & Kennedy, 2010). Better mental health literacy was also characterized as being more likely to give proper advice to psychologically distressed friends, less stigma towards psychiatric illness, and better views about mental health services (Wu et al., 2016). In addition, positive mental health literacy was also associated with better mental health status (Bjørnsen et al., 2019; Lam, 2014).

Considering the impact mental illness could have, it is expected that young people have good mental health literacy. Unfortunately, studies showed that young people have only a moderate level of mental health literacy (Coles et al., 2016; Khan et al., 2010). In a study among 1707 of 12 to 14 years old students, only 3.0% were reported to have adequate mental health literacy (Sarbhan Singh et al., 2020). Similarly, the level of recognition of mental disorder in 325 students was found to be low. Only 27.5% recognized anxiety and 42.4% identified depression (Olsson & Kennedy, 2010). Although it was reported that students had better recognition of depression than anxiety disorder. However, the rate of recognizing depression is still low (Coles et al., 2016; Georgakakou-Koutsonikou & Williams, 2017; Nguyen Thai & Nguyen, 2018; Sarbhan Singh et al., 2019).

In addition, intervention to increase mental health literacy among young people is limited, as most of the studies are conducted among adult (Brijnath, Protheroe, Mahtani, & Antoniades, 2016; Kauer, Mangan, & Sanci, 2014; Lo et al., 2018; Tay et al., 2018). In view of its critical that the issue being tackle during this period (youth), intervention studies that can cater to this specific population should be developed (Xu et al., 2018). Ideally, young people should be able to recognize mental health problems and seek for help when needed. Therefore, this study aims to determine the effectiveness of theory- and web-based health education intervention in increasing mental health literacy among young people.

1.3 Significance of Study

In 2018, there are about 5.5million adolescents in Malaysia (Institute for Public Health, 2017). Youth is a crucial developmental period. Firstly, during this period, brain development and maturation occur through dynamic and highly complex neural remodeling, involving changes in structures and connectivity. Neurodevelopmental changes occurring during youth make it a period of both vulnerability and opportunity for mental health promotion. Social and developmental turmoil occurs during this period as youth try to negotiate several challenges, including transition into multiple social roles from the limited and dependent roles of childhood and simultaneous formation of distinct identities. Second, the period of youth is the starting point of overall mental health issues, as 50% of the onset of mental illness occurs at 14 years old and 75% occurs before 24 years old. Finally, mental health problems during youth have many consequences in later life. Young people are therefore thought to be an important agent of change due to, 1) high prevalence of mental illness, 2) going through a transitional phase and 3) the negative effects it could bring to adult life.

Early detection is one of the important public health strategies. The essential principles of improving solutions for mental health issues for young people need to be based on addressing barriers in accessing mental health services. This is in view of delays in treatment will result in poorer outcomes (Malla et al., 2018). About 25 – 50% of adult mental illnesses may be prevented through early intervention in childhood and adolescence (Kim-Cohen et al., 2003). In addition, the economic benefit of early childhood interventions has been estimated on average to exceed their costs by a ratio of 1:6.

The concepts of health literacy are being used as a guide to mental health literacy and contribute to mental health promotion (World Health Organization, 2005). Mental health literacy is an important componeny of health promotion (Salazar de Pablo et al., 2020). Mental health promotion involves adopting an approach based on positive views of mental health rather than emphasizing mental illness and deficits. Health promotion focuses on enhancing the strengths, capacity, and resources of individuals and communities to enable them to increase control over their mental health and its determinant (Fusar-Poli et al., 2020). The Ottawa Charter of Health Promotion (WHO 1986) provides a foundation for health promotion strategies. Mental health literacy evolved beyond recognition of mental disorders to a more complex construct of health literacy as a social determinant of health, educationally driven intervention with demonstrated positive impact on the health outcomes of individuals and populations, as well as a vehicle that can be applied to help transform health inequities (Kutcher et al., 2016; World Health Organization, 2013b).

WHO asserts that health promotion is a more effective strategy than illness prevention in developing and maintaining population health. Promoting mental health and well-being are an integral part of the sustainable development agenda to transform our world by 2030, as adopted by United Nations General Assembly in 2015. In addition, United Nations has set the suicide rate as one of the indicators in the Sustainable Development Goals with the aim to reduce by one-third premature mortality from non-communicable diseases through prevention and treatment and promote mental health and well-being (United Nations, 2016). In addition, mental health promotion is also one of the objective for Mental Health Action Plan 2013-2020 (World Health Organization, 2013a).

Reviews reported that mental health literacy interventions have been shown to increase literacy particularly on anxiety (Lo et al., 2018; Tay, 2018). In consequence, improvement in mental health literacy will lead to an increase in help-seeking behaviour. Better mental health literacy has also been associated with good mental health status (Bjørnsen et al., 2019; Lam, 2014). Therefore, promotional interventions should include those that support children and young people to develop skills to maintain mental health, that target pre-clinical risk factors, or distress respond to early signs of distress.

One of the recommended strategies for an effective health education intervention is by using theory-based (Walters et al., 2020; Wang et al., 2017; World Health Organization, 2014a). Psychobehavioral theory-based health education intervention allows an understanding of human behaviour. This study will develop health education intervention based on the IMB theory. In addition, it was suggested that promotional

healthy behaviors using psychoeducation and effective theory-based psychological interventions should be increased by leveraging technology (Murray et al., 2017). Technology has become a part of everyday lives, with youth age 15-24 as the most connected age group. It was reported that 80% of the adolescents possess mobile phones and one in three are internet users (UNICEF, 2017). Digital health intervention is, therefore, appeared to be a promising strategy for health education among adolescents. In addition, in view of technology has become ubiquitous in young people's lives, it seems beneficial to incorporate intervention for young people by leveraging technology (Mitchell, 2017).

Unfortunately, despite the importance of promoting good mental health among young people, little empirical research attention is given and interventions for improving mental health among young people are not well established (Fusar-Poli et al., 2020). Furthermore, most of the digital mental health interventions were developed in Western countries and the trials for these programs were also conducted in Western Countries. It is pertinent that mental health education is developed, and its effectiveness is evaluated. Therefore, theory-based health education intervention by leveraging technology in this study is foreseen to increase mental health literacy among young people. By increasing mental health literacy, healthcare would be able to reduce mental health problems by early recognition and intervention. The findings in this study can bring great benefit to the mental health promotion program in Malaysia.

1.4 Research Question

1. Does a theory- and web-based health education intervention effective in improving mental health literacy among foundation students?

2. Does a theory- and web-based health education intervention effective in increasing mental health help seeking behaviour among foundation students?

1.5 Research Objective

1.5.1 General Objective

The general objective for this study is to develop, implement and evaluate the effect of a theory- and web-based health education intervention on mental health literacy among foundation students in UPM.

1.5.2 Specific Objectives

1. To develop a theory- and web-based health education intervention on mental health literacy among foundation students in UPM.

2. To determine and compare baseline of sociodemographic characteristics, mental health literacy, and help-seeking behavior among foundation students in UPM between intervention and control group.

3. To evaluate within and between group effects of a theory- and web-based health education on mental health literacy and help-seeking at 2 weeks post intervention and 1 month follow up.

1.6 Research Hypotheses

1. There is a significance within and between group difference in mental health literacy at baseline, 2 weeks post intervention and 1 month follow up.

2. There is a significance within and between group difference in help-seeking behaviour at 2 weeks post intervention and 1 month follow up.

CHAPTER 2

LITERATURE REVIEW

This chapter presents the information which highlights the definition of depression and anxiety, factors associated with mental disorders, factors associated with mental health literacy, followed by the information motivation behavioral theory. This chapter also provides intervention studies on health education to increase mental health literacy.

2.1 Burden of Mental Disorder

DSM 5 defines mental disorder as a syndrome characterized by clinically significant disturbance in an individual's cognition, emotion regulation, or behavior that reflects a dysfunction in the psychological, biological, or developmental processes underlying mental functioning (Singh, D., & Sinnott-Armstrong, 2015). Common mental disorders (CMD) refer to depressive and anxiety disorders. The prevalence of anxiety and depression is highest among young people and has increased by 60% in the past 25 years. The highest incidence rate of depression is found during adolescence which is alarming. The global prevalence of depression among adolescents was reported to be 25.0% (Silva et al., 2020). Whereas, the 12 months prevalence of depressive disorder among people in their twenties was reported to be 4.4% among men and 7.2% among women respectively (Gustavson et al., 2018). More than 80% of depressive disorders occurred in LMICs. In Malaysia, an overall prevalence of 17.7% depressive symptoms was reported in a study among 24,708 adolescents (Kaur et al., 2014). In another study, the prevalence of depression among 1769 secondary school students was 32.7% (Normala Ibrahim et al., 2017).

Anxiety disorders are among the most common psychiatric conditions, with studies indicating a period prevalence between 9% and 32% during childhood and adolescence (Gustavson et al., 2018). The global prevalence of anxiety disorder among adolescents was reported to be 31.0% (Silva et al., 2020). Whereas, the 12 months prevalence of anxiety disorder among people in their twenties was reported to be 9.6% among men and 26.7% among women respectively (Gustavson et al., 2018). In Malaysia, a 50.8% prevalence of anxiety disorder were reported among 2924 secondary school student (Latiff et al., 2015). In another study among 1860 university students, the prevalence risk on anxiety was 29% (Mohamad et al., 2021).

Mental disorders represent 60-70% of disability-adjusted life years (DALYs) among young people. Mental disorders were the fourth leading cause of DALYs in the Western Pacific in 2000 and reached third place in 2015 (Baranne & Falissard, 2018). Globally, depressive disorders ranked as the single largest contributor to non-fatal health loss, while anxiety disorders ranked as the sixth (World Health Organisation, 2017). Major depressive disorders were also among the five-leading cause of years live with a disability (YLD) in 2016 contributing to 43.1 million of total YLD. Meanwhile, anxiety disorders led to a global total of 24.6 million YLD. Both major depressive disorder and anxiety were also among the main conditions contributing to higher YLD rates in women (Metrics, 2017). In addition, both anxiety and depression were reported to be among the five leading contributors of quality-adjusted life years (QALYs) lost (Abdin et al., 2020).

2.2 Common Mental Disorder

Depression is a condition characterized by persistent unhappiness or a loss of interest or pleasure in most activities. The episode of depression is divided into major and

minor according to the level of the symptoms. According to Diagnostic and Statistical Manual Disorders-5 (DSM 5), a major depressive episode is characterized by a combination of depressed mood or loss of interest or pleasure lasting for most of the day, nearly every day for 2 weeks or more. The primary symptom (depressed mood or loss of interest) must be accompanied by four or more additional symptoms and must cause clinically significant distress or impairment. The primary difference between major depressive episode and major depressive disorder is that major depressive disorder includes all of the criteria for the major depressive episodes as well as its exclusionary criteria of manic and hypomania. The symptoms can be subjectively reported or as observed by others. The American Psychiatric Association has established diagnostic criteria for depressive disorders among youth, as shown in table 2.1.

Table 2.1 Diagnostic Criteria for Depressive Disorder

**A. Depressive Diagnoses**
**Major Depressive Episode:**<br>5 or more of the following A Criteria (at least one includes A1 or A2)<br>A1 Depressed mood or irritability<br>A2 Loss of interest or pleasure in almost all activities<br>A3 Significant (more than 5 percent in a month) unintentional weight loss/ gain or decrease/ increase in appetite.<br>A4 Sleep disturbance (insomnia or hypersomnia)<br>A5 Psychomotor changes (agitation or retardation) severe enough to be observable by others.<br>A6 Tiredness, fatigue, or low energy, or decreased efficiency with which routine tasks are completed.<br>A7 A sense of worthlessness or excessive, inappropriate, or delusional guilt (not merely self-approach or guilt about being sick)<br>A8 Impaired ability to think, concentrate or make decisions.<br>A9 Recurrent thoughts of death (not just fear of dying), suicidal ideation, or suicidal attempts.

Anxiety is a normal human emotion and involves behavioral, affective, and cognitive responses to the perception of anger. It is considered to be excessive or pathological when it is out of proportion to the challenge or stress or when it results in significant distress and impairment (Steimer, 2002). Generalized anxiety disorder (GAD) is an anxiety disorder characterized by excessive anxiety and worry that is not focused on a single trigger. The following anxiety disorder was included in the DSM-5: specific phobia, generalized anxiety disorder, social anxiety disorder, panic disorder, and agoraphobia. Common anxiety disorders among children are specific phobia, social phobia, generalize anxiety disorder, and separation anxiety disorder having mean prevalence rates between 2.2% and 3.6%. Anxiety disorder is also commonly associated with other mental disorders. The correlation between GAD and major depressive disorder was found to be high (Bandelow & Michaelis, 2015). Table 2.2 shows the criteria for GAD according to DSM-5 (Substance Abuse and Mental Health Services Administration, 2016).

Table 2.2 Diagnostic Criteria for Generalized Anxiety Disorder

**Generalized Anxiety Disorder**
A. Excessive anxiety and worry (apprehensive expectation), occurring more days than not for at least 6 months, about a number of events or activities (such as work or school performance)<br><br>B. The person finds it difficult to control the worry.<br><br>C. The anxiety and worry are associated with three or more of the following six symptoms (with at least some symptoms present for more days than not for the past 6 months).<br>Note: Only one item is required in children<br>1. Restlessness or feeling keyed up or on edge.<br>2. Being easily fatigued<br>3. Difficulty concentrating or mind going blank.<br>4. Irritability<br>5. Muscle tension<br>6. Sleep disturbance (difficulty falling or staying asleep, or restless unsatisfying sleep)

D. The anxiety, worry, or physical symptoms cause clinically significant distress or impairment in social, occupational, or other important areas of functioning.

E. The disturbance is not attributable to the physiological effects of a substance (e.g., a drug of abuse, a medication) or another medical condition (e.g., hyperthyroidism).

F. The disturbance is not better explained by another mental disorder (e.g., anxiety or worry about having panic attacks in panic disorder, negative evaluation in social anxiety disorder [social phobia], contamination or other obsessions in obsessive-compulsive disorder, separation from attachment figures in separation anxiety disorder, reminders of traumatic events in post-traumatic stress disorder, gaining weight in anorexia nervosa, physical complaints in somatic symptom disorder, perceived appearance flaws in body dysmorphic disorder, having a serious illness in illness anxiety disorder, or the content of delusional beliefs in schizophrenia or delusional disorder).

2.3 Determinants of Mental Disorder

An interplay of social factors, individual factors, and environmental factors influence the risk of occurrences of mental disorders. These factors influence the life course from before birth until early adulthood towards a person's mental health. For example, substance use during pregnancy and malnutrition can affect mental health during the prenatal period (Colizzi et al., 2020). A multilevel framework was also applied to explain the social determinants that include a life-course approach; community-level contexts including environment and health care systems, and country-level contexts including political and economic factors, cultural norms, and specific policies (Wasserman et al., 2005; World Health Organization, 2014b).

The social determinants framework focuses on understanding how the circumstance in which people live and work shape their health outcomes. Social factors that have been associated with a higher risk of acquiring mental illness include low education, low self-esteem, and low socioeconomic status (Assari, 2017; Blanco et al., 2014; Brown et al., 2017; Othman et al., 2019).

Individual characteristics such as gender and race/ ethnicity are also factors influencing mental health (Alegría et al., 2018). Females are frequently associated with a higher risk of getting the mental illness (Narmandakh et al., 2020). Other individual factors include physical activity (Kim & Kim, 2017; Soltanian et al., 2014), sleep quality (Mohamad et al., 2021), and substance use such as smoking and alcohol (Dooley et al., 2015; Kim & Kim, 2017; Mohamad et al., 2021). Family history of mental disorder also associated with higher risk of getting mental disorder (Dooley et al., 2015; Narmandakh et al., 2020).

A review of environmental factors influencing the mental health of children reported five themes, namely, physical, home, social, socioeconomic, and digital environments. Abuse, either in the form of sexual harassment, physical aggression, or cyberbullying was found as significant sub-theme affecting the mental health of children's and adolescent's mental health (Basu & Banerjee, 2020).

2.4 Impact of Mental Disorder

As described above, depression is a predominant cause of disability for young people. Mental disorders have long-lasting effects on health, social, and economic on individuals, their families, and society (Chen et al., 2006). The contribution of loss of gross domestic product (GDP), resulting from mental disorders and addictions, is reported to be on par with cardiovascular diseases (Trautmann et al., 2016). Individuals with mental disorders also have a shorter life expectancy than the general population, as well as high comorbidity with physical health problems (De Hert et al., 2011).

2.4.1 Adult and Social

Mental disorders will also limit opportunities to lead fulfilling lives as adults. If left untreated, anxiety and depression during youth can predict adult mental health problems. This is because an episode of major depression during youth often leads to chronic or recurrent depression in later life (Gustavson et al., 2018).

Mental disorders can cause prolonged psychosocial, educational, and occupational impairments (Esch et al., 2014; Kessler, 2012). A strong association was reported between mental disorders and education (Esch et al., 2014). It was reported that those with depressive symptoms missed about one day more of school (Glied & Pine, 2002). A major depressive disorder is found to predict significant decrements in role functioning and affecting social life (low marital quality, poor work performance) (Kessler, 2012). Mental disorders particularly depression and anxiety are also among the leading causes of work losses, measured by absenteeism, presenteeism, and sick leave rate (Razzouk, 2017).

2.4.2 Physical Health

Mental health problems are associated with higher risks of getting physical health comorbidities. This is because those with mental illness have higher rates of the risk factors for physical health such as smoking, substance abuse, and nutritional disorders (Lawrence et al., 2009; Minichino et al., 2013; Ohrnberger et al., 2017; Prochaska et al., 2018). This will in turn predispose them to physical health problems like diabetes, cardiovascular and cancer (De Hert et al., 2011).

2.5 Management of Mental Disorder

Treatment of depression and anxiety disorders largely consist of pharmacological treatments, psychological therapies, or a mixture of both. The clinical practice

guideline in Malaysia has recommended psychosocial intervention for mild to moderate major depressive disorder and a combination of pharmacological and psychotherapy for moderate to severe major depressive disorder. Psychoeducation should be offered early and continuously throughout the management of the major depressive disorder.

Psychosocial intervention and psychotherapy that can be offered include but are not restricted to cognitive behavioural therapy, interpersonal therapy, and problem-solving therapy. A combination of pharmacotherapy and psychotherapy should be offered for major depressive disorder. Pharmacological treatment includes selective serotonin uptake inhibitors, noradrenergic norepinephrine reuptake inhibitors, and noradrenergic and specific serotonergic antidepressants should be prescribed in moderate to severe major depressive disorder. Exercise may be offered as an adjunct treatment for moderate to severe depression (Ministry of Health, 2019).

2.5.1 Psychotherapy

Among psychological treatments for depression and anxiety are cognitive behavior therapy (CBT) and interpersonal therapy. CBT has been tailored to target a range of psychiatric disorders including depression, anxiety disorders, bipolar disorder, substance use disorders, personality disorder, and psychotic disorder. The most commonly used intervention for both anxiety and depression among youth and children was based on CBT (Välimäki et al., 2017). CBT is routinely chosen as the psychological treatment of choice and has been subject to several successful trials with moderate to large effect sizes (A. C. James et al., 2018).

CBT is composed of many elements, such as cognitive restructuring, worry exposure, mindfulness, relaxation techniques, and information on the disorder. A comprehensive assessment, development of a good therapeutic relationship and working alliance, cognitive restructuring, repeated exposure with reduction of avoidance behaviour, and skills training comprise the core procedure of CBT for the treatment of anxiety disorders in youth (Seligman & Ollendick, 2011). A review of 137 studies for school-based intervention reported that mindfulness and relaxation-based interventions showed a reduction in anxiety symptoms relative to the usual curriculum (Caldwell et al., 2019).

2.5.2 Pharmacological

Antidepressants such as selective serotonin reuptake inhibitor (SSRI) and serotonin-norepinephrine reuptake inhibitor (SNRI) has been proven effective for depression among young people (Cheung et al., 2018; Ministry of Health, 2019). The most common SSRI are fluoxetine, sertraline, citalopram, paroxetine, duloxetine, and venlafaxine. Among these, fluoxetine has the most evidence to support its use in the adolescent population (Cipriani et al., 2016). Fluoxetine is more efficacious in the treatment of major depressive disorder, whereas fluvoxamine and paroxetine were better in the treatment of anxiety disorder (Boaden et al., 2020). Combination of fluoxetine with CBT was reported to be more effective than CBT alone (Zhou et al., 2020).

2.5.3 Physical Activity

Physical activity is a protective factor for depression and anxiety (Bell et al., 2019; Rosenbaum et al., 2014). Physical activity can decrease the risk for depression by up to 45%, and 60 mins of physical activity each week can prevent 12% of new cases of

depression (World Health Organization, 2019b). Increased self-control and self-esteem, as well as stress resistance, serve as a psychological explanations, while increasing production of neurotransmitters such as dopamine and serotonin are the biological explanations for the positive effects of physical activity on mental health (Lubans et al., 2016).

Physical activity can also reduce depression and anxiety symptoms. Varying intensity of physical activity may lead to a reduction in depression symptoms and that moderate-to vigorous-intensity and light-intensity intervention may reduce anxiety symptoms (Pascoe et al., 2020). A small but significant effect of physical activity on mental health in children and adolescents was reported (Rodriguez-Ayllon et al., 2019). Physical activity was also found to be as effective as CBT or antidepressant medication for mild depressive symptoms (World Health Organization, 2019b). In addition, physical activity was also found to be a cost-effective treatment for anxiety.

2.6 Mental Health Literacy

The development of health literacy was informed by observations that low functional literacy was associated with poorer health outcomes. Health literacy has been defined as ‘the degree to which individuals have the capacity to obtain, process, and understand basic health information and services needed to make appropriate health decisions’. The health literacy concept was primarily focusing on the ability of people to be able to understand and make effective use of medical information, particularly to better understand and better adhere to medication treatments (Cudjoe et al., 2020). Later on, World Health Organizations expands the concept which includes knowledge, skills, and ability of an individual to gain access to, understand and use information in

ways which promote and maintain good health (World Health Organization Regional Office for Europe, 2013).

Health literacy is one of the important parts of health promotion. Health literacy has evolved into a broader construct, which is decreasing health inequities in populations, and enhancing the operation of the health system, and the development of health policy (Mcdaid, 2016; Vamos et al., 2020). In view of, health literacy has been considered as an important component of the social determinants of health, it is, therefore, paramount to consider health literacy to improve the outcome of health (Rowlands et al., 2017). Those with good mental health literacy has also been associated with better mental well-being (Bjørnsen et al., 2019).

Unfortunately, mental health literacy has been ignored in both the health literacy field and the mental health field (Anthony F. Jorm, 2015). Mental illness is often incorrectly perceived as less important than physical health due to its lack of physical symptoms. The World Health Organization (WHO) defines mental health as “a state of well-being in which every individual realizes his or her potential, can cope with the normal stresses of life, can work productively and fruitfully, and is able to contribute to her or his community”. Therefore, the absence of a mental disorder does not necessarily mean the presence of good mental health.

Mental health literacy was first brought into attention in 1997 and was defined by ‘knowledge and beliefs about mental disorders which aid their recognition, management or prevention’ (Anthony F. Jorm et al., 1997). Subsequently, mental health literacy constitutes of six components, namely: 1) knowledge of how to seek

information; 2) knowledge of risk factors and causes; 3) knowledge of self-treatment; 4) knowledge of professional help available; 5) attitudes that promote recognition or appropriate help-seeking behavior; and 6) ability to recognize specific disorders (Anthony F. Jorm et al., 1997). The framework can be found in figure below. Later on, mental health literacy has later been outlined into four key components, namely; 1) understanding how to obtain and maintain good mental health; 2) understanding mental disorders and their treatments; 3) decreasing stigma related to mental disorder, and 4) enhancing help-seeking efficacy (Kutcher et al., 2016).

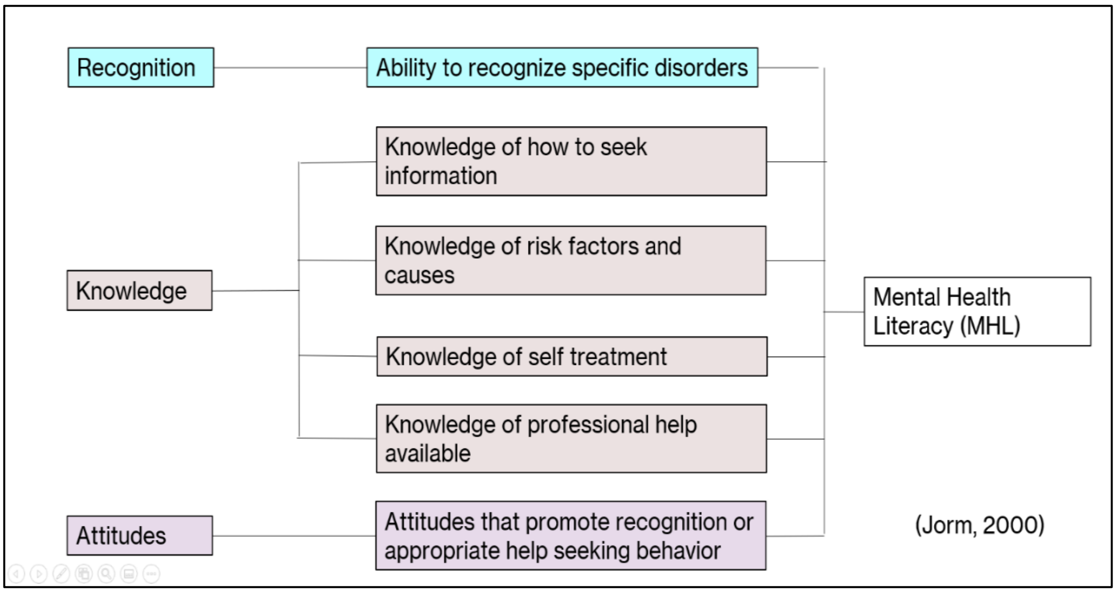

Figure 2.1 Mental Health Literacy Framework

The components of mental health literacy has been identified as factors that can improve access to mental healthcare (Johnson & Possemato, 2019). Early intervention and health promotion are crucial in public health perspective. Health education intervention that incorporates the mental health literacy framework will, therefore, foresee to increase help seeking (Attygalle et al., 2017; Coe, 2009; Newcomb-Anjo, 2019). This is because there is a relationship between health education intervention in

enhancing knowledge and self-efficacy and promoting self-care behavior and health outcomes (Osborn et al., 2011; Squiers et al., 2012).

2.6.1 Factors associated with Mental Health Literacy

In general, the factors of health literacy can be explained by the ecological model, which comprised of individual, interpersonal, and systemic contributors (Manganello, 2008). The factors contributed to mental health literacy can be divided into sociodemographic and individual factors. Sociodemographic factors contributed to mental health literacy are gender, age, level of education and ethnicity. Female, older age and higher level of education are associated with better mental health literacy. Whereas individual factors contributed to mental health literacy are personal experience or family experience of mental illness (Miles et al., 2020). Those with personal experience of depression had better knowledge of the symptoms and therapy (Khan et al., 2010). Similarly, those who have experience with psychological disorders through family or peers have better mental health literacy (Miles et al., 2020).

2.7 Help-seeking

The trajectories of mental disorders can be altered through early recognition and help seeking. Unfortunately, the rate of seeking help for mental health problems among young people is alarmingly low. This is evidenced by about 22.7% of children and adolescents admitted to psychiatric emergency services had no psychiatric diagnosis (Boyer et al., 2013). In a survey, about 35% of young people in the United Kingdom who experienced emotional or mental health difficulties did not seek help. In another study among 619 young people with depression, about 50% had not use any mental health services.

The rate is even higher for the adolescent with suicidality (Cheung & Dewa, 2007). Similarly, it was reported that only 30% of adolescents with mental disorders and 50% with suicidality seek mental health services (Irteja Islam et al., 2020). In addition, despite effective intervention from professional services, it was reported that young people prefer to seek help from family or friends (Coles et al., 2016; Rickwood et al., 2007).

2.7.1 Factors Associated with Help-Seeking

The factors that contributed to help-seeking can be divided into sociodemographic factors, individual factors, social factors, and therapeutic and structural factors (Gulliver et al., 2010; Radez et al., 2020; World Health Organization, 2007). Among these factors, the main determinants of help-seeking are poor health literacy, social stigma, and negative belief towards mental health services (Henderson et al., 2013; Radez et al., 2020; Winter et al., 2017).

Sociodemographic factors

Sociodemographic factors such as gender and socioeconomic status influence help-seeking behavior (Lynch et al., 2018; Magaard et al., 2017; Roberts et al., 2018; Venkataraman et al., 2019). Being female is frequently associated with a higher likelihood of seeking help for mental disorders (Ando et al., 2018; Tan et al., 2020). This could be related to higher self-reliance and personality attribution particularly stigma among males (DeBate et al., 2018).

Individual Factors

Limited mental health literacy is one of the notable barriers to help-seeking (Gulliver et al., 2010; Radez et al., 2020; Rowe et al., 2014). A lack of insight into or understanding the symptoms of mental health were associated with poor help-seeking.

It is also contributed by uncertainty whether their problems were serious or not (Radez et al., 2020). Young people's lack of knowledge about mental health services was also a perceived barrier to help-seeking (Aguirre Velasco et al., 2020). Another individual factor of help-seeking are the preference of self- reliance (Gulliver et al., 2010; Salaheddin & Mason, 2016). Studies indicated that young people prefer to rely on themselves rather than seek external help for their problems. In addition, own perceptions of help-seeking is also a prominent barrier, such as perceived help-seeking as a sign of weakness (Salaheddin & Mason, 2016). Subjective needs also influence help-seeking behavior. Depressive and panic symptoms and the duration of having these symptoms were found to have a positive effect on help-seeking behavior (Nagai, 2015; Roberts et al., 2018).

Social factors

Stigma was cited as the most prominent barrier for help-seeking among young people (Aguirre Velasco et al., 2020; Aldalaykeh et al., 2019; Gulliver et al., 2010; Radez et al., 2020). Most of these were concerns about what others, would think if they were to seek help. In addition, being seen as an "attention seeker" was one of the most relevant obstacles (Rowe et al., 2014). Social support had a positive effect on help-seeking behavior. Family support or belief towards mental health services is also another important barrier related to young people help-seeking (Aguirre Velasco et al., 2020).

The social stigma was defined by Goffman as "an attribute which is deeply discrediting" that reduces a person "from a whole and usual person to a tainted, discounted one". The stigma concept has been reframed for health-related contexts as an adverse social judgement 'based on the enduring feature of identity conferred by a health problem or health-related condition' (Weiss et al., 2006).

People experiencing mental disorders often deal with stigma. This phenomenon includes both cognitive and behavioral aspects. The cognitive aspect comprises stereotypes that are related to knowledge and prejudices which are generalized attitudes towards members of a social group. Whereas discrimination is the behavioral aspect of stigma.

Stigma has many consequences. It can contribute to low self-esteem, reduce the quality of life, as it can have a negative effect on social life, such as work and school, and higher risk of depression. Stigma is also associated with the rejection of help and avoidance of treatment.

Therapeutic and Structural Factors

A major concern of help-seeking behavior of therapeutic factors is the attitude toward mental health services (Aldalaykeh et al., 2019). This can be contributed by the ability to trust healthcare and confidentiality issues (Aguirre Velasco et al., 2020; Radez et al., 2020). Positive past experiences were also associated with the facilitator of help-seeking (Aguirre Velasco et al., 2020). Young people reported that they are more likely to seek help if they feel respected, listened and to not be judged (Radez et al., 2020). A study of factors influencing young people's mental health help-seeking behavior based on health belief model, found that the perceived benefits as the striking factor (P. J. O'Connor et al., 2014). Similarly, perceived effectiveness of professional help was reported to be the reason for not seeking help. Lack of accessibility includes financial costs, time, and logistical barriers were also associated with poor help-seeking. (Aguirre Velasco et al., 2020; Radez et al., 2020; Salaheddin & Mason, 2016).

2.8 Assessment of Mental Health Literacy

Mental Health literacy framework consists of six components and can be categorised into three broad categories namely recognition, knowledge, and attitudes of help seeking. Measurement of mental health literacy should therefore include these three domains. Unfortunately, a systematic literature review of ninety one articles assessing the measurement of mental health literacy revealed conceptual confusion. Most frequent domains that were assesed were recognition and knowledge of mental illnesses (Mansfield et al., 2020). Initially, a “Vignette Interview “ is used to assess mental health literacy. The Vignette Interview described an individual with mental health difficulty and asks aseries of questionss relating to participants uderstanding of what is “wrong” with the individual (Anthony F. Jorm et al., 1997). Although this measure is most extensively used, however, it does not provide a total or subscale score.

Although research on mental health literacy among young people is increasing, however, psychometrically tested measures are not well developed (Mansfield et al., 2020; M. O’Connor et al., 2014). A systematic review of 16 mental health literacy tools in 17 studies reported strong evidence of content validity or internal consistensy in 6 tools (Wei et al., 2016). Only four studies targeting youth. The Mental Health Literacy Scale (MHLS) reported good content vlidity, internal consistensy and reliability. Similarly, the MHLS and The New Inventory Attitudes Toward Seeking Mental Health Services (IASMHS) have good internal consistensy and content validity (Wei et al., 2017). In a recent study evaluating MHLS reported that the scale measured all six attributes of mental health literacy in 29 items (Nejatian et al., 2021).

As for measuring help-seeking, it can be divided into attitude towards help-seeking and intentions to seek help (Divin et al., 2018). The most widely used scales are Attitudes towards Help-Seeking Professional Psychological Help Scale (ATSPPH), the mental health literacy questionnaire (MHLQ) that included items on beliefs towards treatment, the General Help-Seeking Questionnaire (GHSQ) (Wilson et al., 2005), and the Intentions to Seek Counseling Inventory (ISCI) (Divin et al., 2018; Wei et al., 2015).

2.9 Health Behavioral Theories

Theory is defined by a set of interrelated concepts, definitions, and propositions that presents a systematic view of events or situations by specifying relations among variables to explain and predict events or situations. Health behavioral theory helps in understanding the important variables that relate and interacting on behavior (Noar & Zimmerman, 2005). Therefore, health behavioral theories have been recommended as a guide in developing health education intervention (World Health Organization, 2014a). Intervention studies are suggested to manipulate those variables based on the related health behavioral theories (Jeffery, 2004). Example of the health behavioral theories used for health education are the Health Belief Model (HBM), the Theory of Planned Behavior (TPB) and Social Cognitive Theory (SCT) (Glanz et al., 2008).

2.9.1 Social Cognitive Theory

SCT is an interpersonal theory with a core assumption that the interpersonal environment is one of the strongest predictors of health-related behaviour. The SCT has several complex constructs such as reciprocal determinism, environments and situations, observational learning, behavioral capability, reinforcement, outcome expectations, outcome expectancies and self-efficacy. SCT focuses on providing

individuals with the knowledge, skill, or self-confidence to adopt health behaviours. It is therefor, SCT has often used in guiding health literacy interventions. However, due to the complexity of the constructs, application of the SCT often focuses on one or two concepts. Most often used concept is the self-efficacy. There are domains influencing self-efficacy, which are verbal persuasion, emotional arousal, performance accomplishments and vicarious experience (Bandura & Adams, 1977).

2.9.2 Health Belief Model

The HBM consists of six constructs to help predict whether people will take action to prevent, screen for and control illness. The assumptions for HBM are person will take a health-related action based on the feelings of negative health outcome that can be avoided, positive expectation by taking recommended action and believes that recommended health action can be taken. The six constructs are; 1) perceived susceptibility; 2) perceived severity; 3) perceived benefits; 4) perceived barriers; 5) cues to action and 6) self-efficacy(Rosenstock, 1977).

2.9.3 Theory of Planned Behavior

The theory of planned behavior, an extension to the theory of reasoned action will be adopted as the framework to conceptualize the health education intervention. The theory of planned behavior suggests that a person's attitude toward a given behavior, subjective norms concerning the behavior, and perceived control over the behavior are typically good predictors of intention to perform the behavior (Ajzen, 1991).

As mentioned before, this theory focused on the importance of one's beliefs in shaping behavior. According to this theory, an individual's intention in a behaviour comes

from three factors: a) the attitude toward a behaviour, b) subjective norms that stem from social influence, and c) perceived behavioural control.

Attitude describes the extent to which an individual has a positive or negative appraisal toward a specific behaviour. In the context of mental health help-seeking behaviour, attitude focus on the evaluation of behaviour whether seeking help is perceived as being harmful or beneficial. Subjective norms refer to the perceived social pressure of whether to perform or not to perform a behaviour. It includes beliefs about what other people think about behaviour. Therefore, an individual's beliefs about other expectations and the motivation to fulfil them form his or her subjective norms. Perceived behavioural control denotes the extent to which a person perceives behaviour to be performed as easy or difficult. It consists of self-efficacy and controllability, therefore, represents the aspect of ability.

2.9.4 Information Motivation and Behavioral-Skill Theory

The Information Motivation Behavioral skills (IMB) conceptualizes psychological determinants of the performance of behaviors that have the capacity to impair or improve health status. It was originally developed for psychological determinants of HIV risk and preventive behavior. In a systematic review of 12 interventional studies based on IMB, nine studies investigated patients with HIV/ AIDS (Chang et al., 2014).

The IMB model asserts that health-related information, motivation, and behavioral skills are fundamental determinants of health behaviors performance. Individual who are well informed, motivated to act, and possess the requisite behavioral skills will initiate and maintain health promoting behaviors and experience positive health outcomes. Information may include specific facts about health promotion or relevant

heuristics. Motivation is an additional determination that influences whether well-informed individuals will be inclined to undertake health promotion actions. Motivation can be divided into personal (attitude) and social motivation (social support). Whereas, behavioral denotes that a well-informed and well-motivated individual will be capable of enacting the health promotion behaviors (Fisher et al., 2003).

2.9.5 Theoretical Framework

The theoretical framework for this study follows the information motivation and behavioral (IMB) theory. This is in view of six components of mental health literacy framework suit the construct of IMB theory. There was no previous intervention study on mental health literacy that were based on IMB theory. Previous systematic review found that theories that were used for E-health literacy interventions were health belief model (HBM), self-efficacy theory, and a combination of Technology Acceptance Model (TAM) and the Diffusion of Innovations Model (Pourrazavi et al., 2020; Weld et al., 2008). Figure 2.1 below illustrates the theoretical framework for this study based on the IMB theory.

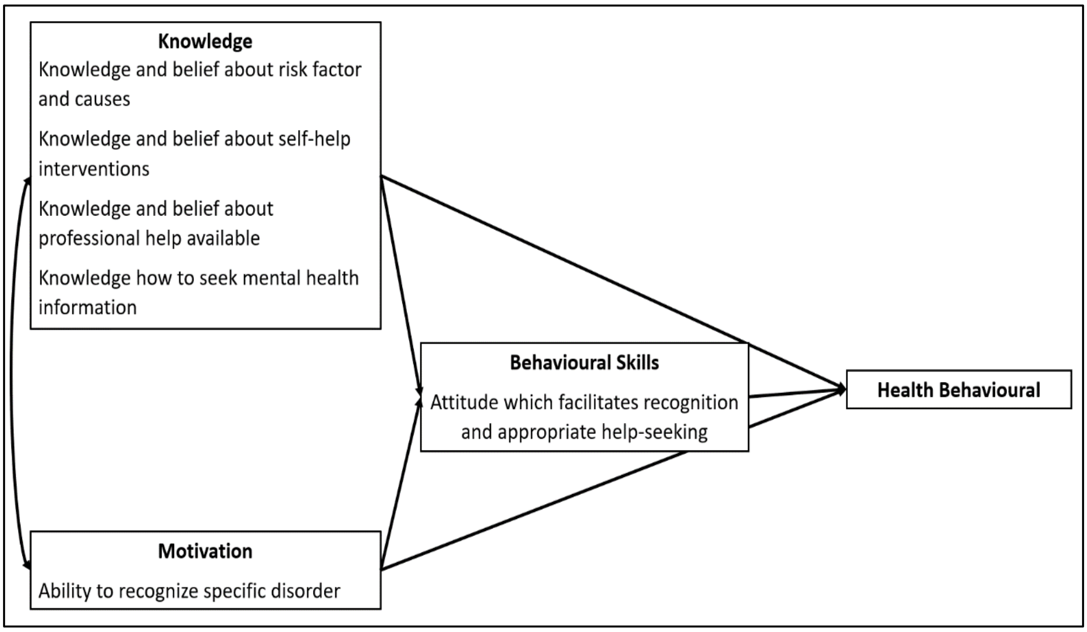

Figure 2.1 Theoretical Framework based on Information Motivation Behavioral Skills Model

2.10 Digital Intervention

It is important to have a delivery platform suitable for the target population (Fullagar et al., 2017). Technology has become ubiquitous in our daily lives, and young people particularly are getting more interested in using an online platforms (Webb et al., 2018). About 90% of smartphone prevalence of ownership in high-income countries and over 50% in low- and middle-income countries were reported among young people.

The umbrella term 'e-mental health' includes a wide range of digitalized services, such as psychoeducational information, electronic patient records, e-learning, screening, counseling, therapy, health promotion, relapse prevention, and self-help. Psychological intervention is considered as digital when technology is used in its delivery, such as the internet, computers, mobile phones, and text messaging services. Modes of delivery for intervention found in engaging young people includes a website, games and computer-assisted programs, apps, robots and digital services, virtual

reality and mobile text messaging (Garrido, Millington, et al., 2019). Young people prefer digital intervention with features such as videos, limited text, the ability to connect with others, and options to receive text message reminders (Liverpool et al., 2020).

Among the barriers for help-seeking in young people are anonymity and poor access. Digital help-seeking tools provide unique characteristics as an important source of support for young people (Stoll et al., 2017; Stunden et al., 2020). This is in view of a digital platform able to connect with others and share experiences while maintaining privacy, immediacy, ease of access, and a greater sense of control over the help-seeking journey (Garrido, Cheers, et al., 2019; Pretorius et al., 2019). The internet may be more commonly used as a place of disclosure rather than asking for help (Mitchell, 2017; Rowe et al., 2014).

Younger patients were found to consult the internet regarding the information on mental illness, psychiatrists, and exchange of information with others (Webelhorst et al., 2020). Young people also showed positive effects on emotional self-awareness using the digital platform (Punukollu & Marques, 2019). Significant improvements in mental health knowledge were also found by using digital intervention (Tay et al., 2018). The most commonly studied digital interventions are Moodgym and Bluepages (Brijnath, Protheroe, Mahtani, Antoniades, et al., 2016; Tay et al., 2018).

2.11 A review on Health Education Interventions to Increase Mental Health Literacy

Health education intervention is fundamental in improving understanding of mental health as part of health promotion (World Health Organization, 2014a). Previous

reviews have shown that health education intervention is effective in increasing mental health literacy and reducing stigma (Brijnath, Protheroe, Mahtani, Antoniades, et al., 2016; Kauer, Mangan, Sanci, et al., 2014; Tay, 2018). However, most of the reviews focused on the adult population (Brijnath, Protheroe, Mahtani, & Antoniades, 2016; Kauer, Mangan, & Sanci, 2014; Tay et al., 2018).

In view of older age can affect mental health literacy, therefore, evidence may not be appliccable to young people. As stigma begins to form and consolidate during youth, and in view of mental disorders are also often emerge during this time, it is therefore important that a tailored health education intervention is developed for young people. Furthermore, previous reviews focused only on the digital platforms (Brijnath, Protheroe, Mahtani, & Antoniades, 2016; Kauer, Mangan, & Sanci, 2014; Tay et al., 2018). Therefore, a review of available intervention on mental health literacy among young people was conducted.

2.11.1 Search Strategy

Searched for the relevant articles on intervention to increase mental health literacy was conducted in two databases: The Cochrane Central Register of Controlled Trials (CENTRAL) and PubMed/MEDLINE up to December 2020. The search was limited to 10 years, from 2010 until 2020. A combination of keywords for mental health literacy OR help-seeking behaviour AND health education AND adolescent OR youth OR young adult OR student was used for the search.

Studies that conducted health education intervention with the outcome to increase mental health literacy targeting depression and anxiety were included. Studies that only have the outcome to reduce stigma or increase help-seeking were excluded.

Studies were included if they were conducted among young people (aged 10-24 years old). Studies that were conducted among adults were excluded. Non-English language studies were excluded. Intervention studies including randomized or quasi clinical trials, cluster-randomized clinical trials were included. Reviews, qualitative studies, descriptive studies, proceedings, and protocol were also excluded.

2.11.2 Baseline Characteristics

Nine studies were found to match the criteria and the findings have been summarised in Table 2.3 through 2.5 (Campos et al., 2018; Norhayati Ibrahim et al., 2020; Li et al., 2013; Milin et al., 2016; Reavley et al., 2014; Skre et al., 2013; Swartz et al., 2017; Taylor-Rodgers & Batterham, 2014; Yamaguchi et al., 2020). Three studies were cluster controlled trial (Reavley et al., 2014; Skre et al., 2013; Yamaguchi et al., 2020), four studies were either quasi or randomized controlled trial (Campos et al., 2018; Milin et al., 2016; Swartz et al., 2017; Taylor-Rodgers & Batterham, 2014), and the remaining one study was a pre-posttest design (Li et al., 2013). The duration of studies was ranging from 2 weeks to 2 years. The percentage of the male was ranging from 25% to 53.9%. Studies were conducted among secondary school students and university students. Baseline characteristics of the studies can be found in Table 2.3.

2.11.3 Intervention (Setting, Mode of Delivery and Content)

Six studies were conducted in a school setting (Campos et al., 2018; Norhayati Ibrahim et al., 2020; Milin et al., 2016; Skre et al., 2013; Swartz et al., 2017; Yamaguchi et al., 2020), two studies were web-based (Reavley et al., 2014; Taylor-Rodgers & Batterham, 2014) and another study were multifaceted interventions (Reavley et al., 2014). In three of the studies that were conducted in a school setting, the intervention was led by the teachers.

The duration of intervention ranging from 50 minutes to three weeks. Interventions were given either through website, talk, video, game and discussion. Interventions were based on education and contact. The content of the health education interventions includes the epidemiology of mental illness (mental illness is common), sign and symptoms of mental illness, stigma reduction, available treatment, and self-help strategies. Most of the studies given the same intervention for the control group after the intervention group had completed (waitlist).

2.11.4 Outcome Measure and Outcome

Most of the studies developed their questionnaire to measure mental health literacy (Campos et al., 2016, 2018; Minichino et al., 2013; Mojtabai et al., 2016; Mukhtar & Oei, 2011; Murray et al., 2017). Two studies used the Depression literacy scale (D-Lit) to measure improvement in literacy (Norhayati Ibrahim et al., 2020; Taylor-Rodgers & Batterham, 2014). As for measuring help-seeking, one study used the Attitude Toward Seeking Professional Help Short Form Scale General Help-Seeking Questionnaire (ATTSPH-SF) to measure help-seeking (Taylor-Rodgers & Batterham, 2014).

Five studies reported a significant increase in mental health literacy after health education intervention (Campos et al., 2018; Norhayati Ibrahim et al., 2020; Li et al., 2013; Swartz et al., 2017; Taylor-Rodgers & Batterham, 2014). Five studies measured help-seeking attitude or behavior (Campos et al., 2018; Norhayati Ibrahim et al., 2020; Reavley et al., 2014; Taylor-Rodgers & Batterham, 2014; Yamaguchi et al., 2020). Out of these five studies, three reported a significant increase in either help-seeking

attitude or behavior (Norhayati Ibrahim et al., 2020; Taylor-Rodgers & Batterham, 2014).

Table 2.3 Baseline Characteristics of Studies

Study	Setting	Study Design	Total Participants (Intervention/ Control)	Duration of study	Mean age	Male (%)	Level of education
**Skre et al., 2013**	School-based (teacher-led)	Non-randomized cluster controlled trial	1070 (520/550)	2 months	I- 14.06 ± 0.85<br>C- 14.29 ± 0.82	53.9%	Secondary School
**Milin et al., 2016**	School-based	RCT	534 (362/ 172)	4-8 weeks	16.5 ± 0.98	44.9%	Secondary School
**Swartz et al., 2017**	School-based (teacher led)	RCT	6679 (3681/ 2998)	4 months	15.8	36%	Secondary School
**Campos et al., 2018**	School-based	RCT	543 (259/284)	6 months	13.04 ± 0.79	52%	Secondary School
**Yamaguchi et al, 2020**	School-based (teacher led)	Quasi-cluster RCT	975 (364/ 611)	2 months	N/A	40.7%	Secondary School
**Ibrahim et al., 2020**	School-based	Quasi-experimental	101 (53/48)	3 months	14.61 ± 1.39	43.8%	Boarding school
**Taylor Rodger et al., 2014**	Web-based	RCT	67 (33/34)	3 weeks	I - 21.9 ± 2.0<br>C- 21.9 ± 1.9	25%	University student
**Reavley et al., 2014**	Web-based	Cluster randomised trial	767 (426/341)	2 years	I- 24.89 ± 8.02<br>C- 23.96 ± 8.89	38%	University student
**Li et al., 2013**	Web-based	Pre/ posttest	73	3 weeks	20.82 ± 1.81	42.5%	Undergraduate students

Abbreviation: RCT- randomized controlled trial, N;/A- not available, I- intervention, C-control

Table 2.4 Characteristics of Intervention

Study	Duration of intervention	Mode of Delivery	Content	Control
**Skre et al., 2013**	3 days	Talk	1. Information about the clinical picture<br>2. Epidemiology<br>3. Treatment of anxiety, depression, eating disorders, and schizophrenia<br>4. Available help system.	Not mentioned
**Milin et al., 2016**	2 weeks	Digital stories, video interview	Curriculum Guide (6 modules)<br>1. Stigma reduction through digital stories and video interview of youth with mental illness<br>2. Understanding of mental health and brain functions<br>3. Mental illnesses and treatment<br>4. Help-seeking behaviors and mental health self-help competencies	Teaching as usual
**Swartz et al., 2017**	3 hours given in 2-3 periods	Interactive lectures, videos, film assignments, homework, and group activities	1. Identifying symptoms of depression<br>2. Understanding the process of medical decision-making<br>3. Seeing parallels between depression and other medical illnesses<br>4. Recognizing suicide as a potential consequence of depression<br>5. Understanding that depression is a treatable medical illness	Waitlist
**Campos et al., 2018**	Two sessions, 90 mins each, one-week interval	Group dynamics, music, and videos.	1. Risk factors for mental health<br>2. Symptoms and signs of five mental disorders<br>3. Promotion of nonstigmatized behaviors towards mental disorders	Not mentioned

			4. Impact of mental health<br>5. Formal and informal help-seeking options<br>6. Promote first aid skills towards people with a mental health problem<br>7. Self-help strategies and explore mental health-promoting behavior.	
**Yamaguchi et al., 2020**	50 minutes	Animated video, discussion	1. Mental disorders being common,<br>2. Mental health problems being closely associated with lifestyle, including sleep habits<br>3. Seeking help from reliable adults being a key to solving the problems<br>4. Knowledge about the symptoms of severe mental illnesses<br>4. Skills to support peers with mental health problems.	Waitlist
**Ibrahim et al., 2020**	Not mentioned	Small group activities, explanatory talk, animation video.	1. Information on mental health and depression<br>2. Facts on mental health statistics	Not mentioned
**Taylor Rodger et al., 2014**	3 weeks		Psychoeducation<br>1. Vignette of a typical young person experiencing a mental health problem<br>2. Description and symptoms<br>3. Challenging stigmatizing views<br>4. Treatment and help options	Link to webpages on dental hygiene, common household medications, and nutrition facts.
**Reavley et al., 2014**	Not mentioned	Website/ Facebook pages, Twitter activities, emails, booklet, posters	1. Depression and related disorders are common in young people<br>2. There are recognizable signs of depression and related disorders in young people<br>3. Early help-seeking leads to better outcomes	Not mentioned

			4. There are several sources of professional help available<br>5. There are useful types of self-help available<br>6. There are helpful first aid actions that staff and peers can take.	
**Li et al., 2013**	3 weeks	Website videogames	“Ching Ching story” (10 topics)<br>1. Identifying stressors and how to handle stress<br>2. Understanding the relationship between stress and coping, and the consequence of depression<br>3. Understanding what goal-directed thinking<br>4. Affirming existing strengths and acknowledging the concept of “self”<br>5. Cognitive restructuring<br>6. Advance cognitive restructuring<br>7. Understanding others’ feelings<br>8. Communication skills<br>9. Conflict resolution based on a problem -solving approach<br>10. Anger management	No control

Table 2.5 Outcome Measure and Outcome

	Outcome Measure	Mental Health Literacy	Help-Seeking
**Skre et al., 2013**	Developed questionnaire on symptom profile recognition, prejudiced belief and knowledge about where to seek help for mental health problems	I- MD 0.27 (0.24-0.30, p<0.001<br>C- MD 0.00 (-0.03, 0.03), p=0.92	Not measured
**Milin et al., 2016**	Developed questionnaire for mental health knowledge and attitude towards mental illness	I- $F_{1,521.74}$ = 20.09, p<0.001<br>C- $F_{1,484.28}$ = 0.55, p-0.459	Not measured
**Swartz et al., 2017**	Developed questionnaire on adolescent depression knowledge and reported and intended behaviour scale	I- aOR 3.10 (2.0,5.0), p<0.001	Not measured
**Campos et al., 2018**	Developed MHLq	5.693 (4.682, 6.704)	0.744 (0.133, 1.356)
**Yamaguchi et al., 2020**	Developed questionnaire on general knowledge about mental health and illness and vignettes	MD 3.92 (3.63, 4.22)	OR=6.32 (3.45,11.74)
**Ibrahim et al., 2020**	D-lit and MHSAS	$F_{(2,84)}$ = 13.266, p<0.001<br>MD 3.233	F (2,84) = 9.939, p<0.001<br>MD 6.535
**Taylor Rodger et al., 2014**	A-Lit, D-Lit, Attitude Toward Seeking Professional Help Short Form Scale General Help-Seeking Questionnaire	Depression literacy- 1.11, p=0.131<br>Anxiety literacy- 3.00, p<0.001	3.46, p= 0.009
**Reavley et al., 2014**	Developed scale based on Vignette	1.56 (0.73-3.33)	1.42 (0.58-3.46)
**Li et al., 2013**	Developed 31 item questionnaires	2.2 (3.66), p<0.001	Not measured

Abbreviation: MHLq- Mental Health Literacy Questionnaire, D-Lit- Depression Literacy scale, MHSAS- Mental Help Seeking Attitude, A-Lit- Anxiety Literature Scale. MD- Mean Difference, aOR- adjusted Odds Ratio.

2.11.5 Summary of Review on Interventions

This review examined the available evidence of health education intervention on mental health literacy and help-seeking behaviour among young people. Nine published studies were identified and included. This review found that most of the studies reported significant improvement in mental health literacy among young people after health education intervention. Out of five studies that assessed the effect on help-seeking behaviour, only two studies reported a significant effect in improving help-seeking behaviour. The findings are similar to previous studies that reported health education interventions only increase mental health literacy, but not help-seeking behaviour (Kauer, Mangan, & Sanci, 2014; Tay et al., 2018).

There are various settings for interventions to improve mental health literacy among young people. This includes whole-of-community interventions, community interventions targeted at young people, school-based interventions, and online-based interventions (Kelly et al., 2007). This review found that most of the health education interventions for young people were conducted in the school setting. The interventions were led by teachers and incorporated into the school curriculum. The school has been shown to be effective as a medium to deliver health education intervention and teachers have emerged as the key (Weare & Nind, 2011). Teachers are an important resource in the implementation of the program, as they already have knowledge and rapport with the children and can adapt to their culture. Furthermore, incorporating the program with the curriculum will ensure the program’s sustainability. However, previous reviews reported inconclusive findings on the effect of a mental health intervention in school (Metrics, 2017; Salerno, 2017; Webelhorst et al., 2020; Wei et al., 2013).

Only a few of the studies were conducted by leveraging technology. Technology has become a part of everyday lives, with youth age 15-24 as the most connected age group. It was reported that 80% of the adolescents possess mobile phones and one in three are internet users

(UNICEF, 2017). Digital health intervention is, therefore, appeared to be a promising strategy for health education among young people. It was suggested that promotional healthy behaviors using psycho-education and effective theory-based psychological interventions should be increased by leveraging technology (Murray et al., 2017). Furthermore, young people are more interested in receiving online health promotion for mental illness (Aschbrenner et al., 2019; Do et al., 2019). Interestingly, the studies that were conducted using web-based only included university students. None of the studies were conducted among school students. The previous reviews on digital health education interventions were also mostly focused on adults (Brijnath, Protheroe, Mahtani, Antoniades, et al., 2016; Xu et al., 2018).

Based on the predictors of help-seeking among young people, strategies to improve help-seeking should focus on improving mental health literacy and reducing stigma (Gulliver et al., 2010). The components of health education intervention should include information on symptoms of mental health problem (such as depression and anxiety), causes of the mental health problem, available mental health services, and treatment available (including pharmacological and non-pharmacological) (Kelly et al., 2007; Okello et al., 2014). The content of the health education intervention in the included studies are the epidemiology of mental illness, sign and symptoms of mental illness, stigma reduction, available treatment and self-help strategies.

Theory of planned behavior constitutes that an individual's intention in a behaviour comes from three factors; namely the attitude toward a behaviour, subjective norms which stem from social influence, and perceived behavioral control. Poor mental health literacy, social stigma, and negative belief towards mental health services and professionals were the most cited predictors for help-seeking behavior. Based on these variables, health education intervention

that aims to increase mental health literacy and reducing stigma appears as promising strategy to promote help-seeking behavior. However, none of the studies developed the intervention based on theory.

In a review of 276 studies, psychoeducation was found to be the most effective intervention in promoting mental health literacy (Salazar de Pablo et al., 2020). A similar finding was reported in a another review (Walters et al., 2020). This is probably because psychoeducational intervention may affect the understanding of depression, symptoms identification, and mental health outcomes (Bevan Jones et al., 2018). However, only one study was reported to give intervention based on psychoeducation (Taylor-Rodgers & Batterham, 2014).

The previous review reported that contents that include active ingredients appear to be more successful (Brijnath, Protheroe, Mahtani, Antoniades, et al., 2016; Tay, 2018). Similarly, previous reviews reported that a delivery method that includes active ingredients appears to be more successful for young people as it increases engagement (Garrido, Millington, et al., 2019; Liverpool et al., 2020; Seedaket et al., 2020; Tay et al., 2018). Interventions were given either through website, talk, video, game, and discussion.

There was heterogeneity in measuring mental health literacy and help-seeking. Although research on mental health literacy among adolescents is increasing, however, psychometrically tested measures are not well developed (Mansfield et al., 2020; M. O'Connor et al., 2014). A systematic review of mental health literacy tools reported that the Mental Health Literacy Scale (MHLS) and The New Inventory Attitudes Toward Seeking Mental Health Services (IASMHS) have good internal consistency and content validity (Wei et al., 2017).

The review founds that health education intervention to increase mental health literacy among young people are mostly conducted in school setting, and digital intervention were moslty conducted among university students. The mode of intervention should be interactive, and the content follows the mental health literacy framework. It would be recommended for future studies to conduct intervention based on theory and assess the outcome using a psychometric tested measure.

2.12 Conceptual Framework

The independent variable for this study will be the mental health education intervention with the primary outcome of mental health literacy and secondary outcome of help seeking behavior. The intervention will be developed based on the IMB theory. Health education intervention will address the knowledge, motivation and behavioral skills of the theory. Besides the construct of the theory, sociodemographic factors such as gender, ethnicity, and socioeconomic status will be included as it also influences mental health literacy (Lynch et al., 2018; Magaard et al., 2017; Venkataraman et al., 2019). Figure 2.2 below illustrates the conceptual framework using the IMB theory for this study.

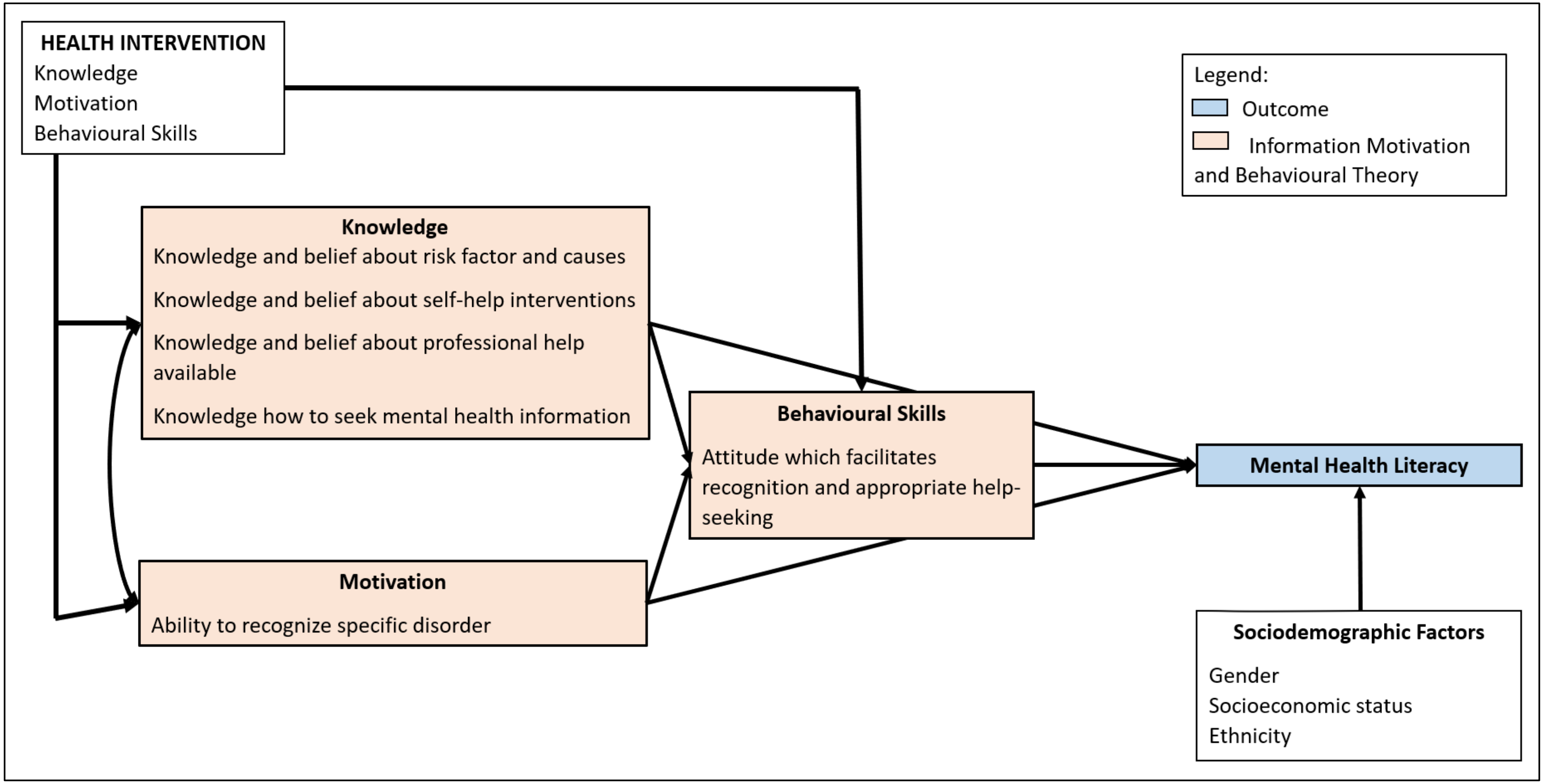

Figure 2.2 Conceptual Framework using Information Motivation Behaviour-Skills Model

CHAPTER 3

METHODOLOGY

This chapter describes the methodology and methods that will be conducted during this study. A detailed explanation that covers the study location, sampling population, sample size, sampling methods, study instrument, intervention program, data analysis strategies, and ethical consideration will be discussed.

3.1 Study Location

The study will be conducted among foundation students at Universiti Putra Malaysia. The program offers a foundation for 'Sijil Pelajaran Malaysia' holders prior to embarking on a degree journey. This foundation program is chosen for the diversity of students enrolled in it, as Malaysian aged 18 and 19 years old, irrespective of ethnicity can apply in the program. The program has two semesters and starts in August every year.

3.2 Study Duration

The study will be conducted over a period of 24 months, from December 2020 until December 2022. The duration of the study comprised of the planning of the research, development of the health education intervention program, implementation of the program, and evaluation of the program. Data collection will commence in September 2021.

3.3 Study Design

This study is a randomized controlled trial study with two arms involving intervention and a control group. Foundation students at University Putra Malaysia will be recruited to join the study. They will be explained about the study objectives, activities that will be required to do if agree for participating. Participants who consented to join the study will then be randomized to either the intervention or control group. Baseline measurement will be taken prior to

intervention. The intervention group will be given a theory-based health education intervention via a newly developed website, whereas the control group will be waitlisted. The intervention will be conducted for two weeks. Measurement will be repeated after the intervention (2 weeks) and at follow-up (1 month). The flow diagram of the study conduct based on the CONSORT 2010 statement (Schulz et al., 2010) is shown in Figure 3.1.

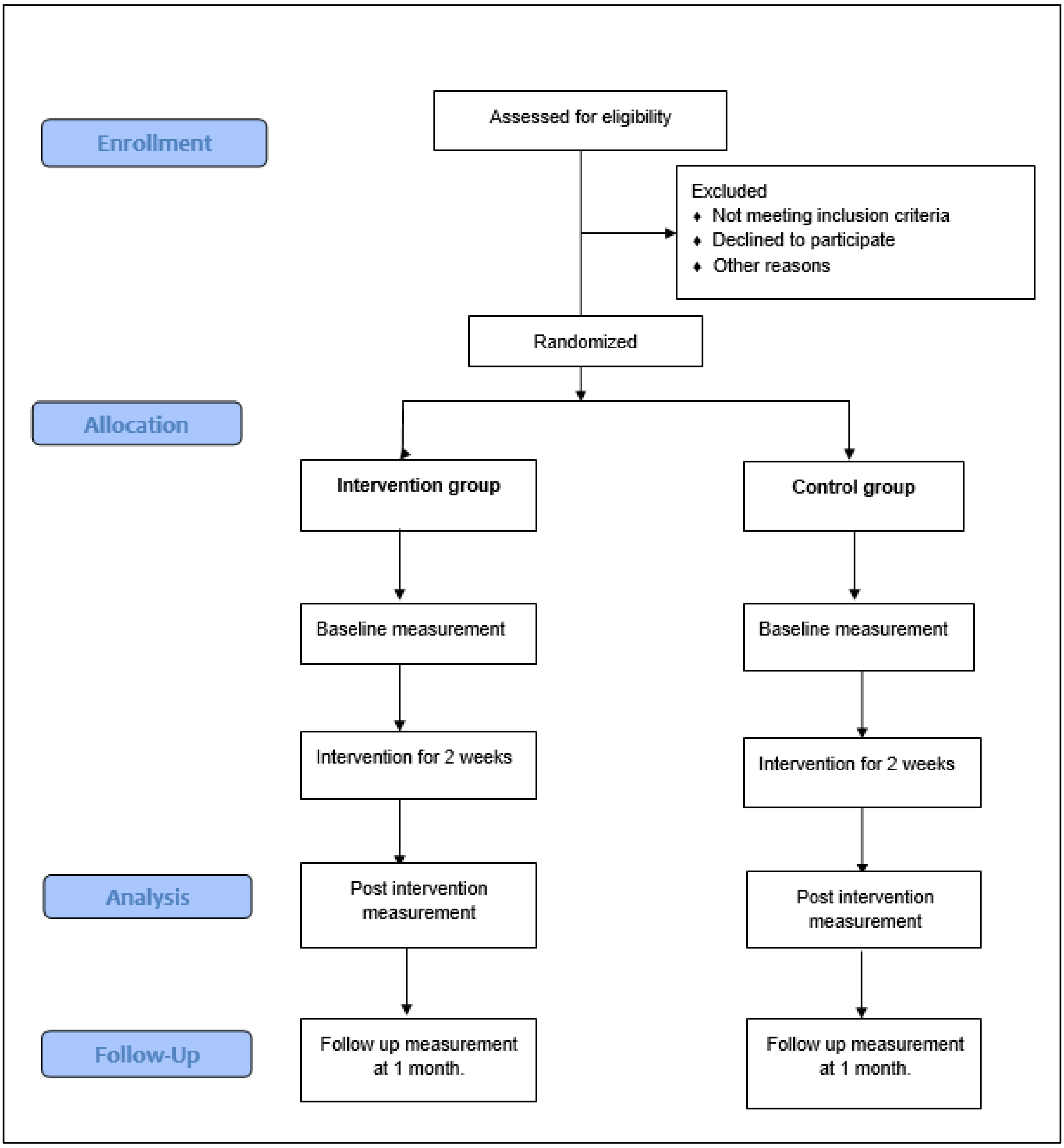

Figure 3.1 Flow Diagram of study Conduct based on CONSORT statement.

3.4 Sampling Method

3.4.1 Study Population

The study population will be foundation students in Universiti Putra Malaysia that met all inclusion and exclusion criteria.

3.4.2 Sampling Population

Foundation students attending Universiti Putra Malaysia.

3.4.3 Selection Criteria

3.4.3.1 Inclusion Criteria

The inclusion criteria in this study are:

i. Malaysian citizen.

ii. Basic skills using digital or computer and internet literate.

iii. Have good command and understanding of the Malay and English language.

3.4.3.2 Exclusion Criteria

The exclusion criteria in this study are:

i. Currently receiving professional assistance for psychiatry diagnosis.

ii. Current severe self-harm or suicidal ideation.

3.4.4 Sampling Frame

List of foundation students attending Universiti Putra Malaysia during the recruitment period.

3.4.5 Sampling Unit

A foundation student attending Universiti Putra Malaysia.

3.4.6 Sampling Method

All the eligible foundation students will be invited to participate in this study. Systematic random sampling will be used to select respondents to be recruited in this study. . Using a sampling interval of 2, every 1 in 2 patients will be selected.

3.4.7 Randomization

Participants who have given consent will be given a unique id number. They will then randomly be allocated to the intervention or control group using their id number. A randomized block design with a block size of four will be used, with the allocation of participants to the group established before the study commenced (Kendall JM, 2003). The randomization will be done by a person who is not a part of the research team, to ensure allocation concealment.

3.4.8 Blinding

A single blinding will we applied involving the participants. Respondents will not be aware of the intervention allocation, but the researcher and those involved in providing the intervention module will be aware of the allocation. Blinding will be ensured by using an encrypted username for the participants. Participants will also be reminded not to share the content of the intervention.

3.4.9 Sample Size

The sample size (n1) for this study was calculated by using the two population mean formula for hypothesis testing (Lemeshow et al., 1990). The sample size calculated was based on the mead Mental Health Literacy Score before and after the intervention. The summary of the sample size calculation is shown in Table 1.

$$N1 = \frac{2SD^2 (z(1-\alpha/2) + z(1-\beta))^2}{(\mu 1-\mu 2)^2}$$

Where,

N = sample size estimate

S = pooled standard deviation

$Z_{1-\alpha/2}$ = 1.96 (confidence level of 95%, α is 0.05)

$Z_{1-\beta}$ = 0.84 (power of 80%, β is 0.2)

$\mu_1-\mu_2$ = estimated mean difference of two groups

S^2 = pooled standard deviation. This is calculated according to IUPAC (IUPAC, 2006) criteria given the equation;

$$S_P = \frac{(n_1-1)s_1^2 + (n_2-1)s_2^2 + \ldots + (n_k - 1)s_k^2}{n_1 + n_2 + n_3 + \ldots + n_k - k}$$

Where;

S_p is the Pooled Standard deviation

n_1 = number of participants in the control group

n_2 = number of participants in the intervention group

s_1 = standard deviation of mental health knowledge in the control group

s_2 = standard deviation of mental health knowledge in the intervention group

Table 3.1 Summary of Sample Size Calculation

References	Mean (sd) intervention group	N intervention group	Mean control group	N control group	Sp	n1
**Campos et al., 2018**	75.91 (8.13)	239	69.02 (8.23)	263	8.18	44

The total sample size in (n_1) in this study is 88 (44 participants per arm). After considering 50% loss to follow up, a final sample size of 132 (66 participants per arm) was determined to be sufficient for this study.

3.5 Study Variables

3.5.1 Dependent Variables

The primary outcome for this study is mental health literacy, and the secondary outcome is help-seeking behaviour. Both the primary and secondary outcomes will be measured at baseline, 2 weeks post-intervention and, 1 month follow-up.

3.5.2 Independent Variable

The independent variable for this study is a health education intervention based on IMB theory delivered via website.

3.5.3 Covariates

The covariates for this study are socio-demographic factors such as age, gender, ethnicity, and household income level.

3.6 Operational Definition

Table 3.2 Operational Definition

Variable	Operational Definition
**Gender**	Gender will be categorized into male and female.
**Ethnicity**	Ethnicity will be categorized into Malay, Chinese, Indian, *Lain-lain*
**Household Income Level**	Household income is combined income for all people sharing a particular household. It will be categorized according to <RM4360, >RM4360 – RM9619, and >RM9619.
**Mental Health Literacy**	Mental Health Literacy will be assessed Mental Health Literacy Scale. It consists of 35 items. The items will be measured using 4-points and 5-points Likert scale. There are six attributes (based on

	the mental health literacy framework). The attributes are; 1) knowledge of where to seek information; 2) ability to recognize disorders; 3) knowledge of self-treatment; 4) knowledges of risk facts and causes; 5) attitudes that promote recognition or appropriate help-seeking behaviour; and 6) knowledge of professional help available. A cumulative mental health literacy score will be calculated.
**Help-Seeking Behavior**	Knowledge of where to access information and capacity to do so. This section consists of 10 items and measured by ‘agree’ or ‘disagree’. The attributes are openness to seeking treatment for emotional problems and values and need in seeking treatment.

3.7 Study Instruments

The study instruments that will be used in this study is validated questionnaire and a health education intervention.

3.7.1 Questionnaire

The questionnaire will be in dual language (English and *Bahasa Melayu*) where the back-to-back translation will be conducted. All parts of the questionnaire will be self–administrated. The questionnaires consist of the following six sections.

To measure the primary outcome, which is mental health literacy, the questionnaire will be adapted from the Mental Health Literacy Scale (MHLS) (M. O’Connor & Casey, 2015). The internal consistency of MHLS were reported to be α=0.8793. Whereas, to measure the secondary outcome which is the help-seeking behaviour, the Attitudes Toward Seeking Professional Psychological Help Scale- Short Form (ATSPPH-SF) (Elhai et al., 2008) will be used. The internal consistency of ATSPPH-SF was reported to be α=0.77. The factor analysis of ASPPH-SF revealed three dimensions includes “openness to seeking professional help”, “value in seeking professional help’, and “preference to cope on one’s own” (Picco et al., 2016).

3.7.1.1 Section One- Sociodemographic

Section one will be sociodemographic factors include age, ethnicity, gender, and household income level.

3.7.1.2 Section Two- Mental Health Literacy

Section two measures mental health literacy using Mental Health Literacy Scale (MHLS) (M. O'Connor & Casey, 2015). This section consists of 35 items. The attributes are the knowledge of where to seek information, ability to recognize disorders, knowledge of self-treatment, knowledges of risk facts and causes, attitudes that promote recognition or appropriate help-seeking behaviour, and knowledge of professional help available. Total score is produced by summing all items. Questions with a 4-point scale are rated 1- very unlikely/unhelpful, 4 – very likely/helpful and for 5-point scale 1 – strongly disagree/definitely unwilling, 5 – strongly agree/definitely willing.

3.7.1.3 Section Four Help-Seeking Behaviour

Section four measures help-seeking behaviour. This section consists of 10 items and measured by 'agree' or 'disagree'. The attributes are openness to seeking treatment for emotional problems and values and need in seeking treatment.

3.7.2 Health Education Intervention

3.7.2.1 Designing and Development of Health Education Intervention Program

The development of the health education module will be going through a few steps. Health intervention module can be developed based on the literature review of previous interventional studies or theory based (Walters et al., 2020; Wang et al., 2017; World Health Organization, 2014a). The development of health education intervention for this study will be based on literature review and the information motivation behavioral theory. The content of the

interventions will be comprised of information on symptoms and causes of mental disorders, available mental health services, pharmacological and non-pharmacological treatment, and self-help strategies. Based on the review, the health education intervention will use active components, to suit the age group (young people) such as video illustrations, quizzes, and vignettes.

The intervention module will then be presented to a panel of experts for content validity. The health education intervention module will also be discussed among the target population (young people) for face validity. This is because it was reported that the involvement of young people during the development of the intervention is recommended. Education intervention with integrating participatory or co-design can increase the effectiveness and sustainability of health education intervention for young people (Bergin et al., 2020; Zorrilla et al., 2019). Figure 3.2 below shows the step of the development of the health education intervention module.

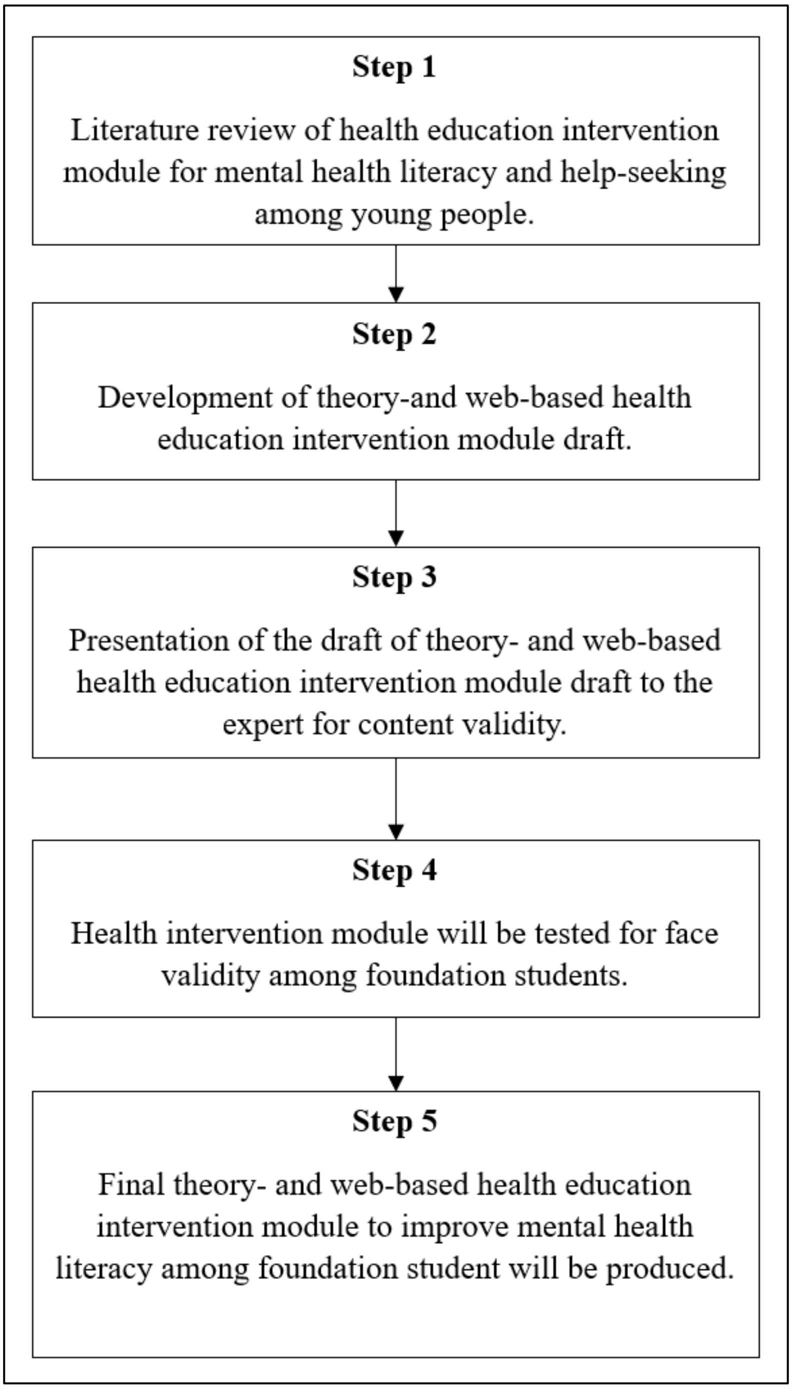

Figure 3.2 Steps of the Development of Health Education Intervention Module

Table 3.3 Structure of the Health Education Intervention Program

Component and Content of Health Education Material	Intervention Group	Control Group (waitlist)	Strategy Delivery	Method of Delivery
**Knowledge**<br>- **Epidemiology**<br>- **Symptoms of anxiety and depression**<br>- **Risk factors of mental disorder**<br>- **How to seek mental health information**<br>- **Self-help intervention**<br>- **Professional help available**	√	√	Website	Video<br>Information<br>Contact
**Motivation**<br>- **Ability to recognise specific disorder**	√	√	Website	Video<br>Information<br>Vignette
**Behavioural Skills**<br>- **Attitude which facilitates recognition and appropriate help-seeking**	√	√	Website	Video<br>Information

3.7.2.2 Implementation of Health Intervention Program

The health education intervention will be implemented after the baseline assessment was conducted. The health education intervention will be given over a period of 2 weeks with 1 month follow-up. The intervention will be done in four sessions, with two sessions weekly. Each session will take approximately 10 minutes to complete.

The participants will be given a link to the website and an id number. The id number will also be their username to access the website of the health education intervention. This is to ensure that non-participants have no access to the education intervention and as monitoring for completion of the intervention. Weekly email and WhatsApp reminders will also be given to participants to prompt them to complete the session.

3.7.2.3 Evaluation of Intervention Program

The outcome of the health education intervention is mental health literacy. Upon completion of the two weeks intervention period, the impact and outcome of the intervention program will be conducted again, using the same set of questionnaires which consists of the measurements for mental health literacy. The evaluations will then made by comparing the result of the pre-intervention with the result of the post-intervention after the health education program was administered. This will be performed for both the intervention and control groups. The evaluation of the health education process will also be done to control group, to assure or improve the quality of the intervention program.

3.8 Quality Control of Study Instruments

3.8.1 Questionnaire

The content validity for the questionnaire will be performed by three panel of experts which include two Public Health Physicians and one Psychologist. The questionnaire will also be pre-tested, and face validated among 30 young people.

3.8.1.1 Content Validity

Content Validity of the questionnaire will be done to assess domain clarity, content relevance, and content representativeness by a panel of expert. Content validity index (CVI) will be calculated for each item. Each item will be assessed by an expert based on the content relevance or representativeness through a 4-point Likert scale ranging from 1 (not relevant) to 4 (extremely relevant). I-CVI will be calculated by counting the number of experts who rated the item as 3 or 4 and dividing by the total number of experts (3) (Almanasreh et al., 2019).

3.8.1.2 Face Validity

Pilot testing will be done to assess the feasibility of the questionnaire and testing the measurement instrument (Abu Hassan et al., 2006). A pilot test will be conducted among a similar populations of interest (university students). This is because we need to ensure that the content is culturally acceptable by the target group. Face validity will be conducted to ensure that it measures the construct is supposed to measure.

3.8.1.3 Reliability

Test-retest reliability will also be conducted during the pre-test to ensure it does that answers from the same respondents on the same measurement are correlated. The intra-class correlation coefficient and Cohen's kappa will be measured. Internal consistency reliability will also be conducted during the pre-test to estimate the degree of consistency among the items. Cronbach alpha will be measured and a value of 0.7 and above will be considered as acceptable reliability.

3.8.2 Health Intervention Module

The content validity for the questionnaire will be performed by three panel of experts which include two Public Health Physicians and one Psychologist. The questionnaire will also be pre-tested, and face validated among 30 young people.

3.8.2.1 Content Validity

The health intervention module will be presented to a panel of experts which consists of two public health physician and one psyschologist. All items which include the theory of planned behaviour-based health education module, videos, content will be presented for the panel to review extensively. All comments and feedback will be taken and the amendment will be done accordingly.

3.8.2.2. Face Validity

The health education intervention will be translated to Malay language and pre-tested among young people to assess their understanding and face validity. The face validity will be measured by asking about the materials, the delivery of health education and if there is any improvement or changes that should be made. All of the comments and feedback will be taken and the amendment will be done accordingly.

3.9 Data Collection

Data will be collected using pre-tested questionnaires. Evaluations will be made at three-time points, once at baseline after consent is taken from participants, following the two weeks after the intervention and at one month follow up. Prior to the commencement of the intervention, a baseline pre-test assessment will be conducted to identify participant's sociodemographic status and mental health literacy. Then the mental health literacy questionnaire will be evaluated post intervention and at follow-up. They will then measure and compared.

3.10 Data Analysis

3.10.1 Descriptive Analysis

Data will be analysed using the IBM Statistical Package for Social Science (SPSS) version 25. Descriptive statistics will be used to describe the baseline characteristics of respondents. Data will be analysed according to intention to treat analysis. Mean and standard deviation will be used for continuous data, the median and interquartile range for not-normally distributed continuous data, and percentage for categorical data. The normality of the data will be check using histogram, Kolmogorov-Smirnov, and Shapiro-Wilk normality test.

3.10.2 Inferential Analysis

The outcome variable will be compared at the baseline and post-test. For within-group difference, if data were assuming normally distributed data, parametric tests such as Paired t-

test will be utilized. But if data were assuming to be non-normal distributed data, non-parametric tests such Wilcoxon Signed Ranks test will utilize. For between groups difference, if data were assumed to be normally distributed data, parametric tests such as Independent t-test will be utilized. But if data were assuming to be non-normally distributed data, non-parametric tests such as the Mann Whitney U test will be utilized. Chi-Square test and Fisher Exact will be used to test the association between two categorical variables.

Multivariable analysis was done using generalized estimating equations (GEE) to determine the effect of the intervention on the primary and secondary outcome after adjusting for the covariates. The effectiveness of the intervention will be based on the trial group and timepoint interaction result. The effect of the intervention on changes in outcome measures will be determined after the intervention period. A summary of the statistical tests that will be used in this study can be found in Table 3.4.

Table 3.4 Summary of Statistical Tests

Objectives	Statistical Tests
**Sociodemographic**	Chi-Square
**Baseline Mental Health Literacy and Help-Seeking Behavior**	Independent t-test
**Post Intervention Mental Health Literacy and Help-Seeking (within group difference)**	Paired t-test
**Post Intervention Mental Health Literacy and Help Seeking (between group difference)**	Independent t-test
**Effect of Health Education Intervention**	Generalized Estimating Equation

3.11 Study Ethics

3.11.1 Ethical Approval

Ethical clearance will be obtained from the following institutions.

i) Ethics Committee for Human Research of the Faculty of Medicine and Health Sciences, Universiti Putra Malaysia.

3.11.2 Consent

Informed consent will be obtained from the respondents prior to data collection.

3.11.3 Data Storage

Study data and personal information will be stored in the researcher's computer. These data will then be transferred to the records and documentation system under the Universiti Putra Malaysia system. The data will then be destroyed five years later. Further to this, any study participant who requested to know their study findings would be informed via email correspondence.

3.11.4 Publication

For publication, all the respondents involved were reported anonymously. This is to protect the confidentiality of the subjects' personal information.

References

Abbafati, C., Machado, D. B., Cislaghi, B., Salman, O. M., Karanikolos, M., McKee, M., Abbas, K. M., Brady, O. J., Larson, H. J., Trias-Llimós, S., Cummins, S., Langan, S. M., Sartorius, B., Hafiz, A., Jenabi, E., Mohammad Gholi Mezerji, N., Borzouei, S., Azarian, G., Khazaei, S., … Zhu, C. (2020). Global burden of 369 diseases and injuries in 204 countries and territories, 1990–2019: a systematic analysis for the Global Burden of Disease Study 2019. *The Lancet*, *396*(10258), 1204–1222. https://doi.org/10.1016/S0140-6736(20)30925-9

Abdin, E., Chong, S. A., Vaingankar, J. A., Shafie, S., Verma, S., Luo, N., Tan, K. B., James, L., Heng, D., & Subramaniam, M. (2020). Impact of mental disorders and chronic physical conditions on quality-adjusted life years in Singapore. *Scientific Reports*, *10*(1), 1–8. https://doi.org/10.1038/s41598-020-59604-0

Abu Hassan, Z., Schattner, P., Mazza, D., Keluarga, K., & Lumpur, K. (2006). DOING A PILOT STUDY: WHY IS IT ESSENTIAL? *Malaysian Family Physician*, *1*(3), 170–173.

Aguirre Velasco, A., Cruz, I. S. S., Billings, J., Jimenez, M., & Rowe, S. (2020). What are the barriers, facilitators and interventions targeting help-seeking behaviours for common mental health problems in adolescents? A systematic review. *BMC Psychiatry*, *20*(1). https://doi.org/10.1186/s12888-020-02659-0

Ahmad, N. A., Cheong, S. M., Ibrahim, N., & Rosman, A. (2014). Suicidal ideation among Malaysian adolescents. *Asia-Pacific Journal of Public Health / Asia-Pacific Academic Consortium for Public Health*, *26*(5 Suppl). https://doi.org/10.1177/1010539514540746

Ajzen, I. (1991). The theory of planned behavior. *Organizational Behavior and Human Decision Processes*, *50*(2), 179–211. https://doi.org/10.1016/0749-5978(91)90020-T

Aldalaykeh, M., Al-Hammouri, M. M., & Rababah, J. (2019). Predictors of mental health services help-seeking behavior among university students. *Cogent Psychology*, *6*(1). https://doi.org/10.1080/23311908.2019.1660520

Alegría, M., NeMoyer, A., Falgàs Bagué, I., Wang, Y., & Alvarez, K. (2018). Social Determinants of Mental Health: Where We Are and Where We Need to Go. *Current Psychiatry Reports*, *20*(11), 1–20. https://doi.org/10.1007/s11920-018-0969-9

Almanasreh, E., Moles, R., & Chen, T. F. (2019). Evaluation of methods used for estimating content validity. *Research in Social and Administrative Pharmacy*, *15*(2), 214–221. https://doi.org/10.1016/j.sapharm.2018.03.066

Altamura, A. C., Santini, A., Salvadori, D., & Mundo, E. (2005). Duration of untreated illness in panic disorder: a poor outcome risk factor? *Neuropsychiatric Disease and Treatment*.

Ando, S., Nishida, A., Usami, S., Koike, S., Yamasaki, S., Kanata, S., Fujikawa, S., Furukawa, T. A., Fukuda, M., Sawyer, S. M., Hiraiwa-Hasegawa, M., & Kasai, K. (2018). Help-seeking intention for depression in early adolescents: Associated factors and sex differences. *Journal of Affective Disorders*, *238*(March), 359–365. https://doi.org/10.1016/j.jad.2018.05.077

Aschbrenner, K. A., Naslund, J. A., Tomlinson, E. F., Kinney, A., Pratt, S. I., & Brunette, M.

F. (2019). Adolescents’ use of digital technologies and preferences for mobile health coaching in public mental health settings. *Frontiers in Public Health*, *7*(JUL), 1–9. https://doi.org/10.3389/fpubh.2019.00178

Assari, S. (2017). Social determinants of depression: The intersections of race, gender, and socioeconomic status. *Brain Sciences*, *7*(12). https://doi.org/10.3390/brainsci7120156

Attygalle, U. R., Perera, H., & Jayamanne, B. D. W. (2017). Mental health literacy in adolescents: Ability to recognise problems, helpful interventions and outcomes. *Child and Adolescent Psychiatry and Mental Health*, *11*(1), 1–8. https://doi.org/10.1186/s13034-017-0176-1

Bandelow, B., & Michaelis, S. (2015). Epidemiology of anxiety disorders in the 21st century. *Dialogues in Clinical Neuroscience*. https://doi.org/10.31887/dcns.2015.17.3/bbandelow

Bandura, A., & Adams, N. E. (1977). Analysis of self-efficacy theory of behavioral change. *Cognitive Therapy and Research*, *1*(4), 287–310. https://doi.org/10.1007/BF01663995

Baranne, M. L., & Falissard, B. (2018). Global burden of mental disorders among children aged 5-14 years. *Child and Adolescent Psychiatry and Mental Health*, *12*(1), 1–9. https://doi.org/10.1186/s13034-018-0225-4

Barker, M. M., Beresford, B., Bland, M., & Fraser, L. K. (2019). Prevalence and Incidence of Anxiety and Depression among Children, Adolescents, and Young Adults with Life-Limiting Conditions: A Systematic Review and Meta-analysis. *JAMA Pediatrics*, *173*(9), 835–844. https://doi.org/10.1001/jamapediatrics.2019.1712

Basu, S., & Banerjee, B. (2020). Impact of environmental factors on mental health of children and adolescents: A systematic review. *Children and Youth Services Review*, *119*(September), 105515. https://doi.org/10.1016/j.childyouth.2020.105515

Bell, S. L., Audrey, S., Gunnell, D., Cooper, A., & Campbell, R. (2019). The relationship between physical activity, mental wellbeing and symptoms of mental health disorder in adolescents: A cohort study. *International Journal of Behavioral Nutrition and Physical Activity*, *16*(1), 1–12. https://doi.org/10.1186/s12966-019-0901-7

Bergin, A. D., Vallejos, E. P., Davies, E. B., Daley, D., Ford, T., Harold, G., Hetrick, S., Kidner, M., Long, Y., Merry, S., Morriss, R., Sayal, K., Sonuga-Barke, E., Robinson, J., Torous, J., & Hollis, C. (2020). Preventive digital mental health interventions for children and young people: a review of the design and reporting of research. *Npj Digital Medicine*, *3*(1). https://doi.org/10.1038/s41746-020-00339-7

Bevan Jones, R., Thapar, A., Stone, Z., Thapar, A., Jones, I., Smith, D., & Simpson, S. (2018). Psychoeducational interventions in adolescent depression: A systematic review. *Patient Education and Counseling*, *101*(5), 804–816. https://doi.org/10.1016/j.pec.2017.10.015

Bilsen, J. (2018). Suicide and Youth: Risk Factors. *Frontiers in Psychiatry*, *9*(October), 1–5. https://doi.org/10.3389/fpsyt.2018.00540

Bjørnsen, H. N., Espnes, G. A., Eilertsen, M. E. B., Ringdal, R., & Moksnes, U. K. (2019). The Relationship Between Positive Mental Health Literacy and Mental Well-Being Among Adolescents: Implications for School Health Services. *Journal of School Nursing*, *35*(2), 107–116. https://doi.org/10.1177/1059840517732125

Blanco, C., Rubio, J., Wall, M., Wang, S., Jiu, C. J., & Kendler, K. S. (2014). Risk factors for

anxiety disorders: Common and specific effects in a national sample. *Depression and Anxiety*. https://doi.org/10.1002/da.22247

Boaden, K., Tomlinson, A., Cortese, S., & Cipriani, A. (2020). Antidepressants in Children and Adolescents: Meta-Review of Efficacy, Tolerability and Suicidality in Acute Treatment. *Frontiers in Psychiatry*, *11*(September), 1–13. https://doi.org/10.3389/fpsyt.2020.00717

Bohon, L. M., Cotter, K. A., Kravitz, R. L., Cello, P. C., & Fernandez y Garcia, E. (2016). The Theory of Planned Behavior as it predicts potential intention to seek mental health services for depression among college students. *Journal of American College Health*, *64*(8), 593–603. https://doi.org/10.1080/07448481.2016.1207646

Boyer, L., Henry, J.-M., Samuelian, J.-C., Belzeaux, R., Auquier, P., Lancon, C., & Da Fonseca, D. (2013). Mental Disorders among Children and Adolescents Admitted to a French Psychiatric Emergency Service. *Emergency Medicine International*, *2013*, 1–7. https://doi.org/10.1155/2013/651530

Brijnath, B., Protheroe, J., Mahtani, K. R., & Antoniades, J. (2016). Do web-based mental health literacy interventions improve the mental health literacy of adult consumers? results from a systematic review. *Journal of Medical Internet Research*, *18*(6), 1–19. https://doi.org/10.2196/jmir.5463

Brijnath, B., Protheroe, J., Mahtani, K. R., Antoniades, J., Sciences, H., Kingdom, U., Kingdom, U., Sciences, H., Brijnath, B., Campus, B., Protheroe, J., & Mahtani, K. R. (2016). *Do Web-based Mental Health Literacy Interventions Improve the Mental Health Literacy of Adult Consumers ? Results From a Systematic Review*. *18*(6), 1–19. https://doi.org/10.2196/jmir.5463

Brown, C. R., Hambleton, I. R., Sobers-Grannum, N., Hercules, S. M., Unwin, N., Nigel Harris, E., Wilks, R., Macleish, M., Sullivan, L., & Murphy, M. M. (2017). Social determinants of depression and suicidal behaviour in the Caribbean: A systematic review. *BMC Public Health*, *17*(1), 1–11. https://doi.org/10.1186/s12889-017-4371-z

Bukh, J. D., Bock, C., Vinberg, M., & Kessing, L. V. (2013). The effect of prolonged duration of untreated depression on antidepressant treatment outcome. *Journal of Affective Disorders*, *145*(1), 42–48. https://doi.org/10.1016/j.jad.2012.07.008

Caldwell, D. M., Davies, S. R., Hetrick, S. E., Palmer, J. C., Caro, P., López-López, J. A., Gunnell, D., Kidger, J., Thomas, J., French, C., Stockings, E., Campbell, R., & Welton, N. J. (2019). School-based interventions to prevent anxiety and depression in children and young people: a systematic review and network meta-analysis. *The Lancet Psychiatry*, *6*(12), 1011–1020. https://doi.org/10.1016/S2215-0366(19)30403-1

Campos, L., Dias, P., Duarte, A., Veiga, E., Dias, C. C., & Palha, F. (2018). Is it possible to “Find space for mental health” in young people? Effectiveness of a school-based mental health literacy promotion program. *International Journal of Environmental Research and Public Health*, *15*(7). https://doi.org/10.3390/ijerph15071426

Campos, L., Dias, P., Palha, F., Duarte, A., & Veiga, E. (2016). Development and psychometric properties of a new questionnaire for assessing mental health literacy in young people. *Universitas Psychologica*, *15*(2), 61–72. https://doi.org/10.11144/Javeriana.upsy15-2.dppq

Chang, S. J., Choi, S., Kim, S. A., & Song, M. (2014). Intervention strategies based on

information-motivation-behavioral skills model for health behavior change: A systematic review. *Asian Nursing Research*, *8*(3), 172–181. https://doi.org/10.1016/j.anr.2014.08.002

Chen, H., Cohen, P., Kasen, S., Johnson, J. G., Berenson, K., & Gordon, K. (2006). Impact of adolescent mental disorders and physical illnesses on quality of life 17 years later. *Archives of Pediatrics and Adolescent Medicine*, *160*(1), 93–99. https://doi.org/10.1001/archpedi.160.1.93

Cheung, A. H., & Dewa, C. S. (2007). Mental health service use among adolescents and young adults with major depressive disorder and suicidality. *Canadian Journal of Psychiatry*, *52*(4), 228–232. https://doi.org/10.1177/070674370705200404

Cheung, A. H., Zuckerbrot, R. A., Jensen, P. S., Laraque, D., & Stein, R. E. K. (2018). Guidelines for adolescent depression in primary care (GLAD-PC): Part II. Treatment and ongoing management. *Pediatrics*, *141*(3). https://doi.org/10.1542/peds.2017-4082

Cipriani, A., Zhou, X., Del Giovane, C., Hetrick, S. E., Qin, B., Whittington, C., Coghill, D., Zhang, Y., Hazell, P., Leucht, S., Cuijpers, P., Pu, J., Cohen, D., Ravindran, A. V., Liu, Y., Michael, K. D., Yang, L., Liu, L., & Xie, P. (2016). Comparative efficacy and tolerability of antidepressants for major depressive disorder in children and adolescents: a network meta-analysis. *The Lancet*, *388*(10047), 881–890. https://doi.org/10.1016/S0140-6736(16)30385-3

Coe, N. (2009). Critical Evaluation of the Mental Health Literacy Framework Using Qualitative Data. *International Journal of Mental Health Promotion*, *11*(4), 34–44. https://doi.org/10.1080/14623730.2009.9721798

Coles, M. E., Ravid, A., Gibb, B., George-Denn, D., Bronstein, L. R., & McLeod, S. (2016). Adolescent Mental Health Literacy: Young People's Knowledge of Depression and Social Anxiety Disorder. *Journal of Adolescent Health*, *58*(1), 57–62. https://doi.org/10.1016/j.jadohealth.2015.09.017

Colizzi, M., Lasalvia, A., & Ruggeri, M. (2020). Prevention and early intervention in youth mental health: Is it time for a multidisciplinary and trans-diagnostic model for care? *International Journal of Mental Health Systems*, *14*(1), 1–14. https://doi.org/10.1186/s13033-020-00356-9

Cudjoe, J., Delva, S., Cajita, M., & Han, H. R. (2020). Empirically Tested Health Literacy Frameworks. *Health Literacy Research and Practice*, *4*(1), e22–e44. https://doi.org/10.3928/24748307-20191025-01

De Hert, M., Correll, C. U., Bobes, J., Cetkovich-Bakmas, M., Cohen, D. A. N., Asai, I., Detraux, J., Gautam, S., Möller, H. J., Ndetei, D. M., Newcomer, J. W., Uwakwe, R., & Leucht, S. (2011). Physical illness in patients with severe mental disorders. I. Prevalence, impact of medications and disparities in health care. *World Psychiatry*, *10*(1), 52–77. https://doi.org/10.1002/j.2051-5545.2011.tb00014.x

DeBate, R. D. G., Gatto, A., & Rafal, G. (2018). The Effects of Stigma on Determinants of Mental Health Help-Seeking Behaviors Among Male College Students: An Application of the Information-Motivation-Behavioral Skills Model. *American Journal of Men's Health*, *12*(5), 1286–1296. https://doi.org/10.1177/1557988318773656

Divin, N., Harper, P., Curran, E., Corry, D., & Leavey, G. (2018). Help-Seeking Measures and Their Use in Adolescents: A Systematic Review. *Adolescent Research Review*, *3*(1),

113–122. https://doi.org/10.1007/s40894-017-0078-8

Do, R., Park, J. R., Lee, S. Y., Cho, M. J., Kim, J. S., & Shin, M. S. (2019). Adolescents' attitudes and intentions toward help-seeking and computer-based treatment for depression. *Psychiatry Investigation*, *16*(10), 728–736. https://doi.org/10.30773/pi.2019.07.17.4

Dooley, B., Fitzgerald, A., & Mac Giollabhui, N. (2015). The risk and protective factors associated with depression and anxiety in a national sample of Irish adolescents. *Irish Journal of Psychological Medicine*, *32*(1), 93–105. https://doi.org/10.1017/ipm.2014.83

Elhai, J. D., Schweinle, W., & Anderson, S. M. (2008). Reliability and validity of the Attitudes Toward Seeking Professional Psychological Help Scale-Short Form. *Psychiatry Research*. https://doi.org/10.1016/j.psychres.2007.04.020

Esch, P., Bocquet, V., Pull, C., Couffignal, S., Lehnert, T., Graas, M., Fond-Harmant, L., & Ansseau, M. (2014). The downward spiral of mental disorders and educational attainment: A systematic review on early school leaving. *BMC Psychiatry*, *14*(1), 1–13. https://doi.org/10.1186/s12888-014-0237-4

Fisher, W. A., Fisher, J. D., & Harman, J. (2003). The Information-Motivation-Behavioral Skills Model: A General Social Psychological Approach to Understanding and Promoting Health Behavior. *Social Psychological Foundations of Health and Illness*, 82–106. https://doi.org/10.1002/9780470753552.ch4

Fu, Z., Burger, H., Arjadi, R., & Bockting, C. L. H. (2020). Effectiveness of digital psychological interventions for mental health problems in low-income and middle-income countries: a systematic review and meta-analysis. *The Lancet Psychiatry*, *7*(10), 851–864. https://doi.org/10.1016/S2215-0366(20)30256-X

Fullagar, S., Rich, E., Francombe-Webb, J., & Maturo, A. (2017). Digital ecologies of youth mental health: Apps, therapeutic publics and Pedagogy as affective arrangements. *Social Sciences*, *6*(4). https://doi.org/10.3390/socsci6040135

Fusar-Poli, P., Salazar de Pablo, G., De Micheli, A., Nieman, D. H., Correll, C. U., Kessing, L. V., Pfennig, A., Bechdolf, A., Borgwardt, S., Arango, C., & van Amelsvoort, T. (2020). What is good mental health? A scoping review. *European Neuropsychopharmacology*, *31*, 33–46. https://doi.org/10.1016/j.euroneuro.2019.12.105

Garrido, S., Cheers, D., Boydell, K., Nguyen, Q. V., Schubert, E., Dunne, L., & Meade, T. (2019). Young People's Response to Six Smartphone Apps for Anxiety and Depression: Focus Group Study. *JMIR Mental Health*, *6*(10), e14385. https://doi.org/10.2196/14385

Garrido, S., Millington, C., Cheers, D., Boydell, K., Schubert, E., Meade, T., & Nguyen, Q. V. (2019). What Works and What Doesn't Work? A Systematic Review of Digital Mental Health Interventions for Depression and Anxiety in Young People. *Frontiers in Psychiatry*, *10*(November), 1–19. https://doi.org/10.3389/fpsyt.2019.00759

Georgakakou-Koutsonikou, N., & Williams, J. M. (2017). Children and young people's conceptualizations of depression: a systematic review and narrative meta-synthesis. *Child: Care, Health and Development*, *43*(2), 161–181. https://doi.org/10.1111/cch.12439

Glanz, K., Rimer, B. k., & Viswanath, K. (2008). *Health Behaviour and Health Education*.

Glied, S., & Pine, D. S. (2002). Consequences and correlates of adolescent depression.

Archives of Pediatrics and Adolescent Medicine, *156*(10), 1009–1014. https://doi.org/10.1001/archpedi.156.10.1009

Gulliver, A., Griffiths, K. M., & Christensen, H. (2010). Perceived barriers and facilitators to mental health help-seeking in young people: A systematic review. *BMC Psychiatry*, *10*. https://doi.org/10.1186/1471-244X-10-113

Gustavson, K., Knudsen, A. K., Nesvåg, R., Knudsen, G. P., Vollset, S. E., & Reichborn-Kjennerud, T. (2018). Prevalence and stability of mental disorders among young adults: Findings from a longitudinal study. *BMC Psychiatry*, *18*(1), 1–15. https://doi.org/10.1186/s12888-018-1647-5

Henderson, C., Evans-Lacko, S., & Thornicroft, G. (2013). Mental illness stigma, help seeking, and public health programs. *American Journal of Public Health*, *103*(5), 777–780. https://doi.org/10.2105/AJPH.2012.301056

Hung, C. I., Liu, C. Y., & Yang, C. H. (2017). Untreated duration predicted the severity of depression at the two-year follow-up point. *PLoS ONE*, *12*(9), 1–11. https://doi.org/10.1371/journal.pone.0185119

Ibrahim, Norhayati, Amit, N., Shahar, S., Wee, L. H., Ismail, R., Khairuddin, R., Siau, C. S., & Safien, A. M. (2019). Do depression literacy, mental illness beliefs and stigma influence mental health help-seeking attitude? A cross-sectional study of secondary school and university students from B40 households in Malaysia. *BMC Public Health*, *19*(Suppl 4), 1–8. https://doi.org/10.1186/s12889-019-6862-6

Ibrahim, Norhayati, Mohd Safien, A., Siau, C. S., & Shahar, S. (2020). The Effectiveness of a Depression Literacy Program on Stigma and Mental Help-Seeking Among Adolescents in Malaysia: A Control Group Study With 3-Month Follow-Up. *Inquiry (United States)*, *57*. https://doi.org/10.1177/0046958020902332

Ibrahim, Normala, Sidik, S. M., Kar, P. C., Mukhtar, F., Awang, H., Kiat, A. J., Osman, Z. J., & Ghaffar, S. F. A. (2017). Prevalence and predictors of depression and suicidal ideation among adolescents attending government secondary schools in Malaysia. *Medical Journal of Malaysia*, *72*(4), 221–227.

Im, Y., Oh, W. O., & Suk, M. (2017). Risk Factors for Suicide Ideation Among Adolescents: Five-Year National Data Analysis. *Archives of Psychiatric Nursing*, *31*(3), 282–286. https://doi.org/10.1016/j.apnu.2017.01.001

Institute for Public Health. (2017). The National Health and Morbidity Survey 2017: Adolescent Health and Nutrition Survey. In *Perpustakaan Negara Malaysia*.

Institute for Public Health Malaysia. (2015). National Health & Morbidity Survey 2015. In *Institute for Public Health, National Institutes of Health, Ministry of Health Malaysia, Kuala Lumpur*.

Institute for Public Health Malaysia. (2019). *Key findings National Health and Morbidity Survey 2019 Non-communicable disease, healthcare demand and health literacy* (Vol. 20). https://doi.org/10.18356/be4d1601-en

Irteja Islam, M., Khanam, R., & Kabir, E. (2020). The use of mental health services by Australian adolescents with mental disorders and suicidality: Findings from a nationwide cross-sectional survey. *PLoS ONE*, *15*(4), 1–17. https://doi.org/10.1371/journal.pone.0231180

James, A. C., Reardon, T., Soler, A., James, G., & Creswell, C. (2018). Cognitive behavioural therapy for anxiety disorders in children and adolescents. *Cochrane Database of Systematic Reviews*, *2018*(10). https://doi.org/10.1002/14651858.CD013162

James, S. L., Abate, D., Abate, K. H., Abay, S. M., Abbafati, C., Abbasi, N., Abbastabar, H., Abd-Allah, F., Abdela, J., Abdelalim, A., Abdollahpour, I., Abdulkader, R. S., Abebe, Z., Abera, S. F., Abil, O. Z., Abraha, H. N., Abu-Raddad, L. J., Abu-Rmeileh, N. M. E., Accrombessi, M. M. K., … Murray, C. J. L. (2018). Global, regional, and national incidence, prevalence, and years lived with disability for 354 Diseases and Injuries for 195 countries and territories, 1990-2017: A systematic analysis for the Global Burden of Disease Study 2017. *The Lancet*, *392*(10159), 1789–1858. https://doi.org/10.1016/S0140-6736(18)32279-7

Jeffery, R. W. (2004). How can Health Behavior Theory be made more useful for intervention research? *International Journal of Behavioral Nutrition and Physical Activity*, *1*, 1–5. https://doi.org/10.1186/1479-5868-1-10

Johnson, E. M., & Possemato, K. (2019). Defining the things we can change to improve access to mental health care. *Families, Systems & Health : The Journal of Collaborative Family Healthcare*, *37*(3), 195–205. https://doi.org/10.1037/fsh0000435

Jorm, A. F. (2000). Mental health literacy: Public knowledge and beliefs about mental disorders. In *British Journal of Psychiatry*. https://doi.org/10.1192/bjp.177.5.396

Jorm, Anthony F. (2015). Why We Need the Concept of “Mental Health Literacy.” *Health Communication*, *30*(12), 1166–1168. https://doi.org/10.1080/10410236.2015.1037423

Jorm, Anthony F., Korten, A. E., Jacomb, P. A., Christensen, H., Rodgers, B., & Pollitt, P. (1997). “Mental health literacy”: A survey of the public’s ability to recognise mental disorders and their beliefs about the effectiveness of treatment. *Medical Journal of Australia*. https://doi.org/10.5694/j.1326-5377.1997.tb140071.x

Jung, H., von Sternberg, K., & Davis, K. (2017). The impact of mental health literacy, stigma, and social support on attitudes toward mental health help-seeking. *International Journal of Mental Health Promotion*, *19*(5), 252–267. https://doi.org/10.1080/14623730.2017.1345687

Kauer, S. D., Mangan, C., & Sanci, L. (2014). Do online mental health services improve help-seeking for young people? a systematic review. *Journal of Medical Internet Research*, *16*(3), 1–25. https://doi.org/10.2196/jmir.3103

Kauer, S. D., Mangan, C., Sanci, L., Foundation, T. I., Kauer, S. D., & Mangan, C. (2014). *Do Online Mental Health Services Improve Help-Seeking for Young People ? A Systematic Review*. *16*(3), 1–25. https://doi.org/10.2196/jmir.3103

Kaur, J., Cheong, S. M., Mahadir Naidu, B., Kaur, G., Manickam, M. A., Mat Noor, M., Ibrahim, N., & Rosman, A. (2014). Prevalence and correlates of depression among adolescents in Malaysia. *Asia-Pacific Journal of Public Health / Asia-Pacific Academic Consortium for Public Health*, *26*(5 Suppl). https://doi.org/10.1177/1010539514544356

Kelly, C. M., Jorm, A. F., & Wright, A. (2007). *Improving mental health literacy as as strategy to facilitate early intervention for mental disorders*. *187*(7), 1–5.

Kendall JM. (2003). Designing a research project: randomised controlled trials and their

principles. *Emergency Medicine Journal*, 164–168. http://search.ebscohost.com/login.aspx?direct=true&AuthType=ip,shib&db=rzh&AN=106870387&site=eds-live&custid=s4165981

Kessler, R. C. (2012). The costs of depression. *Psychiatric Clinics of North America*, *35*(1), 1–14. https://doi.org/10.1016/j.psc.2011.11.005

Kessler, R. C., Amminger, G. P., Aguilar-Gaxiola, S., Alonso, J., Lee, S., & Üstün, T. B. (2007). Age of onset of mental disorders: A review of recent literature. *Current Opinion in Psychiatry, 20*(4), 359–364. https://doi.org/10.1097/YCO.0b013e32816ebc8c

Khan, T. M., Sulaiman, S. A., & Hassali, M. A. (2010). Mental health literacy towards depression among non-medical students at a Malaysian university. *Mental Health in Family Medicine, 7*(1), 27–35.

Kim-Cohen, J., Caspi, A., Moffitt, T. E., Harrington, H., Milne, B. J., & Poulton, R. (2003). Prior Juvenile Diagnoses in Adults With Mental Disorder. *Archives of General Psychiatry*, *60*(7), 709. https://doi.org/10.1001/archpsyc.60.7.709

Kim, J., & Kim, H. (2017). Demographic and environmental factors associated with mental health: A cross-sectional study. *International Journal of Environmental Research and Public Health*, *14*(4). https://doi.org/10.3390/ijerph14040431

Kotera, Y., Ting, S. H., & Neary, S. (2021). Mental health of Malaysian university students: UK comparison, and relationship between negative mental health attitudes, self-compassion, and resilience. *Higher Education*, *81*(2), 403–419. https://doi.org/10.1007/s10734-020-00547-w

Kutcher, S., Wei, Y., Costa, S., Gusmão, R., Skokauskas, N., & Sourander, A. (2016). Enhancing mental health literacy in young people. *European Child and Adolescent Psychiatry*, *25*(6), 567–569. https://doi.org/10.1007/s00787-016-0867-9

Lam, L. T. (2014). Mental health literacy and mental health status in adolescents: A population-based survey. *Child and Adolescent Psychiatry and Mental Health*, *8*(1), 1–8. https://doi.org/10.1186/1753-2000-8-26

Latiff, L. A., Sidik, S. M., Ibrahim, N., & Othman, N. (2015). *Prevalence of anxiety and its association with socio-demographic factors among secondary school students in Pasir Gudang district, Johor Reliability of BACS-M View project Invivo and invitro studies on postmenopausal-induced rats fed with (Nigella sativa)*. *2*(6), 104–112. https://www.researchgate.net/publication/288436752

Lawrence, D., Mitrou, F., & Zubrick, S. R. (2009). Smoking and mental illness: Results from population surveys in Australia and the United States. *BMC Public Health*, *9,* 1–14. https://doi.org/10.1186/1471-2458-9-285

Lemeshow, S., Hosmer Jr, D. W., Klar, J., & Lwanga, S. K. (1990). Adequacy of Sample Size Determination in Health Studies. In *Adequacy of Sample Size in Health Studies*.

Li, T. M. H., Chau, M., Wong, P. W. C., Lai, E. S. Y., & Yip, P. S. F. (2013). Evaluation of a web-based social network electronic game in enhancing mental health literacy for young people. *Journal of Medical Internet Research*, *15*(5), 1–20. https://doi.org/10.2196/jmir.2316

Liverpool, S., Mota, C. P., Sales, C. M. D., Čuš, A., Carletto, S., Hancheva, C., Sousa, S., Cerón, S. C., Moreno-Peral, P., Pietrabissa, G., Moltrecht, B., Ulberg, R., Ferreira, N., &

Edbrooke-Childs, J. (2020). Engaging children and young people in digital mental health interventions: Systematic review of modes of delivery, facilitators, and barriers. *Journal of Medical Internet Research*, *22*(6). https://doi.org/10.2196/16317

Lo, K., Gupta, T., & Keating, J. L. (2018). Interventions to Promote Mental Health Literacy in University Students and Their Clinical Educators. A Systematic Review of Randomised Control Trials. *Health Professions Education*, *4*(3), 161–175. https://doi.org/10.1016/j.hpe.2017.08.001

Lubans, D., Richards, J., Hillman, C., Faulkner, G., Beauchamp, M., Nilsson, M., Kelly, P., Smith, J., Raine, L., & Biddle, S. (2016). Physical activity for cognitive and mental health in youth: A systematic review of mechanisms. *Pediatrics*, *138*(3). https://doi.org/10.1542/peds.2016-1642

Lynch, L., Long, M., & Moorhead, A. (2018). Young Men, Help-Seeking, and Mental Health Services: Exploring Barriers and Solutions. *American Journal of Men's Health*, *12*(1), 138–149. https://doi.org/10.1177/1557988315619469

Magaard, J. L., Seeralan, T., Schulz, H., & Brütt, A. L. (2017). Factors associated with help-seeking behaviour among individuals with major depression: A systematic review. *PLoS ONE*, *12*(5), 1–17. https://doi.org/10.1371/journal.pone.0176730

Malaysian Healthcare Performance Unit. (2017). Malaysian mental healthcare performance. *Malaysian Journal of Psychiatry*, *20*(Editorial), 1–3. http://www.moh.gov.my/moh/resources/Penerbitan/Laporan/Umum/Mental Healthcare Performance Report 2016.pdf%0Ahttp://www.mjpsychiatry.org/index.php/mjp/article/viewFile/147/122%0Afile:///C:/Users/Prof Muhaya/Downloads/147-530-1-PB.pdf

Malla, A., Shah, J., Iyer, S., Boksa, P., Joober, R., Andersson, N., Lal, S., & Fuhrer, R. (2018). Youth Mental Health Should Be a Top Priority for Health Care in Canada. *Canadian Journal of Psychiatry*, *63*(4), 216–222. https://doi.org/10.1177/0706743718758968

Manganello, J. A. (2008). Health literacy and adolescents: A framework and agenda for future research. *Health Education Research*, *23*(5), 840–847. https://doi.org/10.1093/her/cym069

Mansfield, R., Patalay, P., & Humphrey, N. (2020). A systematic literature review of existing conceptualisation and measurement of mental health literacy in adolescent research: Current challenges and inconsistencies. *BMC Public Health*, *20*(1), 1–14. https://doi.org/10.1186/s12889-020-08734-1

Mcdaid, D. (2016). *Investing in health literacy What do we know about the co-benefits to the education sector of actions targeted at children and young people? HEALTH SYSTEMS AND POLICY ANALYSIS*. 25. http://www.euro.who.int/__data/assets/pdf_file/0006/315852/Policy-Brief-19-Investing-health-literacy.pdf?ua=1

Metrics, G. H. (2017). *Global , regional , and national incidence , prevalence , and years lived with disability for 328 diseases and injuries for 195 countries , 1990 – 2016 : a systematic analysis for the Global Burden of Disease Study 2016*. *390*, 1990–2016. https://doi.org/10.1016/S0140-6736(17)32154-2

Miles, R., Rabin, L., Krishnan, A., Grandoit, E., & Kloskowski, K. (2020). Mental health

literacy in a diverse sample of undergraduate students: demographic, psychological, and academic correlates. *BMC Public Health, 20*(1), 1–13. https://doi.org/10.1186/s12889-020-09696-0

Milin, R., Kutcher, S., Lewis, S. P., Walker, S., Wei, Y., Ferrill, N., & Armstrong, M. A. (2016). Impact of a Mental Health Curriculum on Knowledge and Stigma among High School Students: A Randomized Controlled Trial. *Journal of the American Academy of Child and Adolescent Psychiatry, 55*(5), 383-391.e1. https://doi.org/10.1016/j.jaac.2016.02.018

Minichino, A., Bersani, F. S., Calò, W. K., Spagnoli, F., Francesconi, M., Vicinanza, R., Chiaie, R. D., & Biondi, M. (2013). Smoking behaviour and mental health disorders-mutual influences and implications for therapy. *International Journal of Environmental Research and Public Health, 10*(10), 4790–4811. https://doi.org/10.3390/ijerph10104790

Ministry of Health, M. (2019). Management of major depressive disorder (MDD). *Malaysian Family Physician, 6*(1), 13.

Mitchell, C. (2017). *Mental health help-seeking behaviours in young adults*. *January*, 11–12. https://doi.org/10.3399/bjgp17X688453

Mohamad, N. E., Sidik, S. M., Akhtari-Zavare, M., & Gani, N. A. (2021). The prevalence risk of anxiety and its associated factors among university students in Malaysia: a national cross-sectional study. *BMC Public Health, 21*(1), 1–12. https://doi.org/10.1186/s12889-021-10440-5

Mojtabai, R., Olfson, M., & Han, B. (2016). National trends in the prevalence and treatment of depression in adolescents and young adults. *Pediatrics, 138*(6). https://doi.org/10.1542/peds.2016-1878

Mukhtar, F., & Oei, T. P. S. (2011). A review on assessment and treatment for depression in malaysia. *Depression Research and Treatment, 2011*. https://doi.org/10.1155/2011/123642

Murray, E., Edin, F., & Collins, L. M. (2017). *Evaluating digital health intervention: key questions and approaches*. *51*(5), 843–851. https://doi.org/10.1016/j.amepre.2016.06.008.Evaluating

Nagai, S. (2015). Predictors of help-seeking behavior: Distinction between help-seeking intentions and help-seeking behavior. *Japanese Psychological Research, 57*(4), 313–322. https://doi.org/10.1111/jpr.12091

Narmandakh, A., Roest, A. M., de Jonge, P., & Oldehinkel, A. J. (2020). Psychosocial and biological risk factors of anxiety disorders in adolescents: a TRAILS report. *European Child and Adolescent Psychiatry*. https://doi.org/10.1007/s00787-020-01669-3

Nejatian, M., Tehrani, H., Momeniyan, V., & Jafari, A. (2021). A modified version of the mental health literacy scale (MHLS) in Iranian people. *BMC Psychiatry, 21*(1), 1–11. https://doi.org/10.1186/s12888-021-03050-3

Newcomb-Anjo, S. E. (2019). Applying What is Known About Adolescent Development to Improve School-Based Mental Health Literacy of Depression Interventions: Bridging Research to Practice. *Adolescent Research Review, 4*(3), 235–248. https://doi.org/10.1007/s40894-018-0083-6

Nguyen Thai, Q. C., & Nguyen, T. H. (2018). Mental health literacy: Knowledge of depression among undergraduate students in Hanoi, Vietnam. *International Journal of Mental Health Systems*, *12*(1), 1–8. https://doi.org/10.1186/s13033-018-0195-1

Nicole Racine, Jessica E. Cooke, R. E., & Daphne J. Korczak, BraeAnne McArthura, S. M. (2020). Child and adolescent mental illness during COVID-19: A rapid review. *Psychiatry Research*, *January*.

Noar, S. M., & Zimmerman, R. S. (2005). Health Behavior Theory and cumulative knowledge regarding health behaviors: Are we moving in the right direction? *Health Education Research*, *20*(3), 275–290. https://doi.org/10.1093/her/cyg113

O'Connor, M., & Casey, L. (2015). The Mental Health Literacy Scale (MHLS): A new scale-based measure of mental health literacy. *Psychiatry Research*, *229*(1–2), 511–516. https://doi.org/10.1016/j.psychres.2015.05.064

O'Connor, M., Casey, L., & Clough, B. (2014). Measuring mental health literacy-a review of scale-based measures. *Journal of Mental Health*, *23*(4), 197–204. https://doi.org/10.3109/09638237.2014.910646

O'Connor, P. J., Martin, B., Weeks, C. S., & Ong, L. (2014). Factors that influence young people's mental health help-seeking behaviour: A study based on the Health Belief Model. *Journal of Advanced Nursing*, *70*(11), 2577–2587. https://doi.org/10.1111/jan.12423

Ohrnberger, J., Fichera, E., & Sutton, M. (2017). The relationship between physical and mental health: A mediation analysis. *Social Science and Medicine*, *195*(October), 42–49. https://doi.org/10.1016/j.socscimed.2017.11.008

Okello, E. S., Abbo, C., Muhwezi, W. W., Akello, G., & Ovuga, E. (2014). Mental health literacy among secondary school students in North and Central Uganda: a qualitative study. *World Cultural Psychiatry Research Review : Official Journal of World Association of Cultural Psychiatry*, *9*(3), 70–80. http://www.ncbi.nlm.nih.gov/pubmed/26113883%0Ahttp://www.pubmedcentral.nih.gov/articlerender.fcgi?artid=PMC4477034

Olsson, D. P., & Kennedy, M. G. (2010). Mental health literacy among young people in a small US town: Recognition of disorders and hypothetical helping responses. *Early Intervention in Psychiatry*, *4*(4), 291–298. https://doi.org/10.1111/j.1751-7893.2010.00196.x

Osborn, C. Y., Paasche-Orlow, M. K., Bailey, S. C., & Wolf, M. S. (2011). The mechanisms linking health literacy to behavior and health status. *American Journal of Health Behavior*, *35*(1), 118–128. https://doi.org/10.5993/AJHB.35.1.11

Othman, N., Ahmad, F., El Morr, C., & Ritvo, P. (2019). Perceived impact of contextual determinants on depression, anxiety and stress: A survey with university students. *International Journal of Mental Health Systems*, *13*(1), 1–9. https://doi.org/10.1186/s13033-019-0275-x

Pascoe, M., Bailey, A. P., Craike, M., Carter, T., Patten, R., Stepto, N., & Parker, A. (2020). Physical activity and exercise in youth mental health promotion: A scoping review. *BMJ Open Sport and Exercise Medicine*, *6*(1), 1–11. https://doi.org/10.1136/bmjsem-2019-000677

Patton, G., & Temmerman, M. (2016). Evidence and Evidence Gaps in Adolescent Health. *Journal of Adolescent Health*, *59*(2), S1–S3. https://doi.org/10.1016/j.jadohealth.2016.08.001

Picco, L., Abdin, E., Chong, S. A., Pang, S., Shafie, S., Chua, B. Y., Vaingankar, J. A., Ong, L. P., Tay, J., & Subramaniam, M. (2016). Attitudes toward seeking professional psychological help: Factor structure and socio-demographic predictors. *Frontiers in Psychology*, *7*(APR), 1–10. https://doi.org/10.3389/fpsyg.2016.00547

Polanczyk, G. V., Salum, G. A., Sugaya, L. S., Caye, A., & Rohde, L. A. (2015). Annual research review: A meta-analysis of the worldwide prevalence of mental disorders in children and adolescents. *Journal of Child Psychology and Psychiatry and Allied Disciplines*, *56*(3), 345–365. https://doi.org/10.1111/jcpp.12381

Pourrazavi, S., Kouzekanani, K., Bazargan-Hejazi, S., Shaghaghi, A., Hashemiparast, M., Fathifar, Z., & Allahverdipour, H. (2020). Theory-based E-health literacy interventions in older adults: A systematic review. *Archives of Public Health*, *78*(1), 1–8. https://doi.org/10.1186/s13690-020-00455-6

Pretorius, C., Chambers, D., & Coyle, D. (2019). Young people's online help-seeking and mental health difficulties: Systematic narrative review. *Journal of Medical Internet Research*, *21*(11), 1–27. https://doi.org/10.2196/13873

Prochaska, J. J., Das, S., & Young-wolff, K. C. (2018). *Smoking, Mental Illness, and Public Health*. 165–185. https://doi.org/10.1146/annurev-publhealth-031816-044618.Smoking

Punukollu, M., & Marques, M. (2019). Use of mobile apps and technologies in child and adolescent mental health: A systematic review. *Evidence-Based Mental Health*, *22*(4), 161–166. https://doi.org/10.1136/ebmental-2019-300093

Raaj, S., Navanathan, S., Tharmaselan, M., & Lally, J. (2021). Mental disorders in Malaysia: an increase in lifetime prevalence. *BJPsych International*, 1–3. https://doi.org/10.1192/bji.2021.4

Radez, J., Reardon, T., Creswell, C., Lawrence, P. J., Evdoka-Burton, G., & Waite, P. (2020). Why do children and adolescents (not) seek and access professional help for their mental health problems? A systematic review of quantitative and qualitative studies. *European Child and Adolescent Psychiatry*. https://doi.org/10.1007/s00787-019-01469-4

Razzouk, D. (2017). Mental health economics: The costs and benefits of psychiatric care. *Mental Health Economics: The Costs and Benefits of Psychiatric Care*, 1–459. https://doi.org/10.1007/978-3-319-55266-8

Reavley, N. J., McCann, T. V., Cvetkovski, S., & Jorm, A. F. (2014). A multifaceted intervention to improve mental health literacy in students of a multicampus university: a cluster randomised trial. *Social Psychiatry and Psychiatric Epidemiology*, *49*(10), 1655–1666. https://doi.org/10.1007/s00127-014-0880-6

Rickwood, D. J., Deane, F. P., & Wilson, C. J. (2007). When and how do young people seek professional help for mental health problems? In *The Medical journal of Australia*. https://doi.org/10.5694/j.1326-5377.2007.tb01334.x

Roberts, T., Miguel Esponda, G., Krupchanka, D., Shidhaye, R., Patel, V., & Rathod, S. (2018). Factors associated with health service utilisation for common mental disorders: A systematic review. *BMC Psychiatry*, *18*(1), 1–19. https://doi.org/10.1186/s12888-018-

1837-1

Rodriguez-Ayllon, M., Cadenas-Sánchez, C., Estévez-López, F., Muñoz, N. E., Mora-Gonzalez, J., Migueles, J. H., Molina-García, P., Henriksson, H., Mena-Molina, A., Martínez-Vizcaíno, V., Catena, A., Löf, M., Erickson, K. I., Lubans, D. R., Ortega, F. B., & Esteban-Cornejo, I. (2019). Role of Physical Activity and Sedentary Behavior in the Mental Health of Preschoolers, Children and Adolescents: A Systematic Review and Meta-Analysis. *Sports Medicine*, *49*(9), 1383–1410. https://doi.org/10.1007/s40279-019-01099-5

Rosenbaum, S., Tiedemann, A., Sherrington, C., Curtis, J., & Ward, P. B. (2014). Physical activity interventions for people with mental illness: A systematic review and meta-analysis. *Journal of Clinical Psychiatry*. https://doi.org/10.4088/JCP.13r08765

Rosenstock, I. M. (1977). The Health Belief Model and Preventive Health Behavior. *Health Education & Behavior*, *2*(4), 354–386. https://doi.org/10.1177/109019817400200405

Rowe, S. L., French, R. S., Henderson, C., Ougrin, D., Slade, M., & Moran, P. (2014). Help-seeking behaviour and adolescent self-harm: A systematic review. *Australian and New Zealand Journal of Psychiatry*, *48*(12), 1083–1095. https://doi.org/10.1177/0004867414555718

Rowlands, G., Shaw, A., Jaswal, S., Smith, S., & Harpham, T. (2017). Health literacy and the social determinants of health: A qualitative model from adult learners. *Health Promotion International*, *32*(1), 130–138. https://doi.org/10.1093/heapro/dav093

Salaheddin, K., & Mason, B. (2016). Identifying barriers to mental health help-seeking among young adults in the UK:a cross-sectional survey. *British Journal of General Practice*, *66*(651), e686–e692. https://doi.org/10.3399/bjgp16X687313

Salam, R. A., Das, J. K., Lassi, Z. S., & Bhutta, Z. A. (2016). Adolescent Health Interventions: Conclusions, Evidence Gaps, and Research Priorities. *Journal of Adolescent Health*, *59*(2), S88–S92. https://doi.org/10.1016/j.jadohealth.2016.05.006

Salazar de Pablo, G., De Micheli, A., Nieman, D. H., Correll, C. U., Kessing, L. V., Pfennig, A., Bechdolf, A., Borgwardt, S., Arango, C., van Amelsvoort, T., Vieta, E., Solmi, M., Oliver, D., Catalan, A., Verdino, V., Di Maggio, L., Bonoldi, I., Vaquerizo-Serrano, J., Baccaredda Boy, O., … Fusar-Poli, P. (2020). Universal and selective interventions to promote good mental health in young people: Systematic review and meta-analysis. *European Neuropsychopharmacology*, *41*, 28–39. https://doi.org/10.1016/j.euroneuro.2020.10.007

Salerno, J. P. (2017). *review*. *86*(12), 922–931. https://doi.org/10.1111/josh.12461.Effectiveness

Schulz, K. F., Altman, D. C., & Moher, D. (2010). CONSORT 2010 Statement: Updated guidelines for reporting parallel group randomised trials. *Italian Journal of Public Health*. https://doi.org/10.4178/epih/e2014029

Seedaket, S., Turnbull, N., Phajan, T., & Wanchai, A. (2020). Improving mental health literacy in adolescents: systematic review of supporting intervention studies. *Tropical Medicine and International Health*, *25*(9), 1055–1064. https://doi.org/10.1111/tmi.13449

Seligman, L. D., & Ollendick, T. H. (2011). Cognitive-Behavioral Therapy for Anxiety

Disorders in Youth. In *Child and Adolescent Psychiatric Clinics of North America*. https://doi.org/10.1016/j.chc.2011.01.003

Silva, S. A., Silva, S. U., Ronca, D. B., Gonçalves, V. S. S., Dutra, E. S., & Carvalho, K. M. B. (2020). Common mental disorders prevalence in adolescents: A systematic review and metaanalyses. *PLoS ONE*, *15*(4), 1–19. https://doi.org/10.1371/journal.pone.0232007

Singh, D., & Sinnott-Armstrong, W. (2015). *THE DSM-5 DEFINITION OF MENTAL DISORDER*. Public Affairs Quarterly. https://www.jstor.org/stable/43574513?seq=1

Singh, Sarbhan, Zaki, R. A., & Farid, N. D. N. (2019). A systematic review of depression literacy: Knowledge, help-seeking and stigmatising attitudes among adolescents. *Journal of Adolescence*, *74*(June), 154–172. https://doi.org/10.1016/j.adolescence.2019.06.004

Singh, Sarbhan, Zaki, R. A., & Farid, N. D. N. (2020). Adolescent mental health literacy and its association with depression. *ASM Science Journal*, *13*(Specialissue5), 207–216.

Singh, Shweta, Roy, D., Sinha, K., Parveen, S., Sharma, G., & Joshi, G. (2020). Impact of COVID-19 and lockdown on mental health of children and adolescents : A narrative review with recommendations. *Psychiatry Research*, *January*.

Skre, I., Friborg, O., Breivik, C., Johnsen, L. I., Arnesen, Y., & Wang, C. E. A. (2013). A school intervention for mental health literacy in adolescents: Effects of a non-randomized cluster controlled trial. *BMC Public Health*, *13*(1). https://doi.org/10.1186/1471-2458-13-873

Soltanian, A. R., Amiri, M., Namazi, S., Qaedi, H., & Kohan, G. R. (2014). Mental health changes and its predictors in adolescents using the path analytic model: A 7-year observational study. *Iranian Journal of Psychiatry*, *9*(1), 1–7.

Squiers, L., Peinado, S., Berkman, N., Boudewyns, V., & McCormack, L. (2012). The health literacy skills framework. *Journal of Health Communication*, *17*(SUPPL. 3), 30–54. https://doi.org/10.1080/10810730.2012.713442

Steimer, T. (2002). The biology of fear- and anxiety-related behaviors. *Dialogues in Clinical Neuroscience*, *4*(3), 231–249.

Stoll, R. D., Pina, A. A., Gary, K., & Amresh, A. (2017). Usability of a Smartphone Application to Support the Prevention and Early Intervention of Anxiety in Youth. *Cognitive and Behavioral Practice*, *24*(4), 393–404. https://doi.org/10.1016/j.cbpra.2016.11.002

Stunden, C., Zasada, J., VanHeerwaarden, N., Hollenberg, E., Abi-Jaoudé, A., Chaim, G., Cleverley, K., Henderson, J., Johnson, A., Levinson, A., Lo, B., Robb, J., Shi, J., Voineskos, A., & Wiljer, D. (2020). Help-seeking behaviors of transition-aged youth for mental health concerns: Qualitative study. *Journal of Medical Internet Research*, *22*(10). https://doi.org/10.2196/18514

Substance Abuse and Mental Health Services Administration. (2016). DSM-5 Changes: Implications for Child Serious Emotional Disturbance. *DSM-5 Changes: Implications for Child Serious Emotional Disturbance*, *June*, 48. www.ncbi.nlm.nih.gov/books/NBK519712/table/ch3.t8/

Swartz, K., Musci, R. J., Beaudry, M. B., Heley, K., Miller, L., Alfes, C., Townsend, L.,

Thornicroft, G., & Wilcox, H. C. (2017). School-based curriculum to improve depression literacy among US secondary school students: A randomized effectiveness trial. *American Journal of Public Health*, *107*(12), 1970–1976. https://doi.org/10.2105/AJPH.2017.304088

Tan, G. T. H., Shahwan, S., Goh, C. M. J., Ong, W. J., Samari, E., Abdin, E., Kwok, K. W., Chong, S. A., & Subramaniam, M. (2020). Causal beliefs of mental illness and its impact on help-seeking attitudes: a cross-sectional study among university students in Singapore. *BMJ Open*, *10*(7), e035818. https://doi.org/10.1136/bmjopen-2019-035818

Tay, J. L. (2018). *Effectiveness of information and communication technologies interventions to increase mental health literacy : A systematic review*. *May*, 1024–1037. https://doi.org/10.1111/eip.12695

Tay, J. L., Tay, Y. F., & Klainin-Yobas, P. (2018). Effectiveness of information and communication technologies interventions to increase mental health literacy: A systematic review. *Early Intervention in Psychiatry*, *12*(6), 1024–1037. https://doi.org/10.1111/eip.12695

Taylor-Rodgers, E., & Batterham, P. J. (2014). Evaluation of an online psychoeducation intervention to promote mental health help seeking attitudes and intentions among young adults: Randomised controlled trial. *Journal of Affective Disorders*, *168*, 65–71. https://doi.org/10.1016/j.jad.2014.06.047

Thai, T. T., Ly, N., Thi, L., Han, V., & Thi, H. (2020). Mental Health Literacy and Help - Seeking Preferences in High School Students in Ho Chi Minh City , Vietnam. *School Mental Health*. https://doi.org/10.1007/s12310-019-09358-6

Trautmann, S., Rehm, J., & Wittchen, H. U. (2016). The economic costs of mental disorders: Do our societies react appropriately to the burden of mental disorders? *EMBO Reports*, *17*(9), 1245–1249. https://doi.org/10.15252/embr.201642951

UNICEF. (2017). State of the Worlds Children 2017 - Children in a Digital World. In *Unicef*. https://www.unicef.org/publications/index_101992.html

United Nations. (2016). Transforming Our World the 2030 Agenda for Sustainable Development. *Arsenic Research and Global Sustainability - Proceedings of the 6th International Congress on Arsenic in the Environment, AS 2016*, 12–14. https://doi.org/10.1201/b20466-7

Välimäki, M., Anttila, K., Anttila, M., & Lahti, M. (2017). Web-Based Interventions Supporting Adolescents and Young People With Depressive Symptoms: Systematic Review and Meta-Analysis. *JMIR MHealth and UHealth*, *5*(12), e180. https://doi.org/10.2196/mhealth.8624

Vamos, S., Okan, O., Sentell, T., & Rootman, I. (2020). Making a case for "education for health literacy": An international perspective. *International Journal of Environmental Research and Public Health*, *17*(4), 1–18. https://doi.org/10.3390/ijerph17041436

Venkataraman, S., Patil, R., & Balasundaram, S. (2019). Why mental health literacy still matters: a review. *International Journal Of Community Medicine And Public Health*, *6*(6), 2723. https://doi.org/10.18203/2394-6040.ijcmph20192350

Walters, R., Leslie, S. J., Polson, R., Cusack, T., & Gorely, T. (2020). Establishing the efficacy of interventions to improve health literacy and health behaviours: A systematic

review. *BMC Public Health*, *20*(1), 1–17. https://doi.org/10.1186/s12889-020-08991-0

Wang, Y., Fadhil, A., Lange, J. P., & Reiterer, H. (2017). Towards a holistic approach to designing theory-based mobile health interventions. *ArXiv*.

Wasserman, D., Cheng, Q., & Jiang, G.-X. (2005). Global suicide rates among young people aged 15-19. *World Psychiatry : Official Journal of the World Psychiatric Association (WPA)*.

Weare, K., & Nind, M. (2011). Mental health promotion and problem prevention in schools: What does the evidence say? *Health Promotion International*, *26*(SUPPL. 1). https://doi.org/10.1093/heapro/dar075

Webb, C. A., Rosso, I. M., & Rauch, S. L. (2018). *Internet-based Cognitive Behavioral Therapy for Depression: Current Progress & Future Directions*. *25*(3), 114–122. https://doi.org/10.1097/HRP.0000000000000139.Internet-based

Webelhorst, C., Jepsen, L., & Rummel-Kluge, C. (2020). Utilization of e-mental-health and online self-management interventions of patients with mental disorders-A cross-sectional analysis. *PLoS ONE*, *15*(4), 1–15. https://doi.org/10.1371/journal.pone.0231373

Wei, Y., Hayden, J. A., Kutcher, S., Zygmunt, A., & McGrath, P. (2013). The effectiveness of school mental health literacy programs to address knowledge, attitudes and help seeking among youth. *Early Intervention in Psychiatry*, *7*(2), 109–121. https://doi.org/10.1111/eip.12010

Wei, Y., McGrath, P. J., Hayden, J., & Kutcher, S. (2015). Mental health literacy measures evaluating knowledge, attitudes and help-seeking: A scoping review. *BMC Psychiatry*, *15*(1). https://doi.org/10.1186/s12888-015-0681-9

Wei, Y., McGrath, P. J., Hayden, J., & Kutcher, S. (2016). Measurement properties of tools measuring mental health knowledge: A systematic review. *BMC Psychiatry*, *16*(1). https://doi.org/10.1186/s12888-016-1012-5

Wei, Y., McGrath, P. J., Hayden, J., & Kutcher, S. (2017). Measurement properties of mental health literacy tools measuring help-seeking: a systematic review*. *Journal of Mental Health*, *26*(6), 543–555. https://doi.org/10.1080/09638237.2016.1276532

Weiss, M. G., Ramakrishna, J., & Somma, D. (2006). Health-related stigma: Rethinking concepts and interventions. *Psychology, Health and Medicine*, *11*(3), 277–287. https://doi.org/10.1080/13548500600595053

Weld, K. K., Padden, D., Ramsey, G., Garmon Bibb, S. C., & Bibb, S. C. G. (2008). A Framework for Guiding Health Literacy Research in Populations With Universal Access to Healthcare. *Advances in Nursing Science*, *31*(4), 308–318. https://doi.org/10.1097/01.ANS.0000341411.25048.91

Wilson, C. J., Deane, F. P., Ciarrochi, J., & Rickwood, D. (2005). *Measuring Help-Seeking Intentions : Properties of the General Help-Seeking Questionnaire*. *39*, 15–28.

Winter, R. I., Patel, R., & Norman, R. I. (2017). A Qualitative Exploration of the Help-Seeking Behaviors of Students Who Experience Psychological Distress Around Assessment at Medical School. *Academic Psychiatry*, *41*(4), 477–485. https://doi.org/10.1007/s40596-017-0701-9

World Health Organisation. (2017). Depression and other common mental disorders: global health estimates. *World Health Organization*, 1–24.

World Health Organization. (2004). The global burden of disease 2004. *Update, World Health Organization*, 146.

World Health Organization. (2005). Promoting Mental Health. *The Handbook of Community Mental Health Nursing*, 149–163. https://doi.org/10.5840/ncbq201616462

World Health Organization. (2007). Adolescents , social support and help-seeking behaviour consultation with recommendations for action. *World Health Organisation*, 1–64.

World Health Organization. (2013a). *Mental health action plan 2013 - 2020*. https://www.who.int/publications/i/item/9789241506021

World Health Organization. (2013b). The Solid Facts Health Literacy. In *BMJ (Clinical research ed.)*.

World Health Organization. (2014a). Health Education: Theoretical Concepts, Effective Strategies and Core Competencies. In *Health Promotion Practice* (Vol. 15, Issue 5). https://doi.org/10.1177/1524839914538045

World Health Organization. (2014b). *Social determinants of mental health*. 54.

World Health Organization. (2019a). *Fact Sheet: Adolescent Mental disorders*. WHO.

World Health Organization. (2019b). Motion for your mind. *World Health Organization*. http://www.euro.who.int/pubrequest

World Health Organization. (2021). *Adolescent health*. https://www.who.int/southeastasia/health-topics/adolescent-health

World Health Organization Regional Office for Europe. (2013). The Solid Facts Health Literacy. In *BMJ (Clinical research ed.)*.

Wu, C., Lee, M., & Fang, C. (2016). *Factors Associated with Depression-Related Mental Health Literacy in Patients who Attend Emergency Services in*. *1*, 1–8.

Xu, Z., Huang, F., Kösters, M., Staiger, T., Becker, T., Thornicroft, G., & Rüsch, N. (2018). Effectiveness of interventions to promote help-seeking for mental health problems: Systematic review and meta-analysis. *Psychological Medicine*, *48*(16), 2658–2667. https://doi.org/10.1017/S0033291718001265

Yamaguchi, S., Ojio, Y., Foo, J. C., Michigami, E., Usami, S., Fuyama, T., Onuma, K., Oshima, N., Ando, S., Togo, F., & Sasaki, T. (2020). A quasi-cluster randomized controlled trial of a classroom-based mental health literacy educational intervention to promote knowledge and help-seeking/helping behavior in adolescents. *Journal of Adolescence*, *82*(November 2019), 58–66. https://doi.org/10.1016/j.adolescence.2020.05.002

Zhou, X., Teng, T., Zhang, Y., Del Giovane, C., Furukawa, T. A., Weisz, J. R., Li, X., Cuijpers, P., Coghill, D., Xiang, Y., Hetrick, S. E., Leucht, S., Qin, M., Barth, J., Ravindran, A. V., Yang, L., Curry, J., Fan, L., Silva, S. G., … Xie, P. (2020). Comparative efficacy and acceptability of antidepressants, psychotherapies, and their combination for acute treatment of children and adolescents with depressive disorder: a systematic review and network meta-analysis. *The Lancet Psychiatry*, *7*(7), 581–601.

https://doi.org/10.1016/S2215-0366(20)30137-1

Zorrilla, M. M., Modeste, N., Gleason, P. C., Sealy, D. A., Banta, J. E., & Trieu, S. L. (2019). Depression and Help-Seeking Intention Among Young Adults: The Theory of Planned Behavior. *American Journal of Health Education*, *50*(4), 236–244. https://doi.org/10.1080/19325037.2019.1616014
